# Mutations in *KMT2C*, *BCOR* and *KDM5C* Predict Response to Immune Checkpoint Blockade Therapy in Non-Small Cell Lung Cancer

**DOI:** 10.3390/cancers14112816

**Published:** 2022-06-06

**Authors:** Dingxie Liu, Jonathan Benzaquen, Luc G. T. Morris, Marius Ilié, Paul Hofman

**Affiliations:** 1 Bluewater Biotech LLC, New Providence, NJ 07974, USA; 2 Department of Pneumology, Pasteur Hospital, FHU OncoAge, 06000 Nice, France; benzaquen.j@chu-nice.fr (J.B.); ilie.m@chu-nice.fr (M.I.); 3 Department of Surgery, Memorial Sloan Kettering Cancer Center, New York, NY 10065, USA; morrisl@mskcc.org; 4 Laboratory of Clinical and Experimental Pathology, CHU Nice, FHU OncoAge, University Côte d’Azur, 06100 Nice, France; 5 Team 4, IRCAN, UMR 7284/U10181, FHU OncoAge, University Côte d’Azur, 06107 Nice, France; 6 Hospital-Integrated Biobank (BB-0033-00025), CHU of Nice, FHU OncoAge, University Côte d’Azur, 06100 Nice, France

**Keywords:** chromatin remodeling, immune checkpoint blockade, response prediction, non-small cell lung cancer, tumor mutation burden

## Abstract

**Simple Summary:**

Efficient biomarkers are urgently needed to predict response to immune checkpoint blockade (ICB) therapy for non-small cell lung cancer (NSCLC), particularly NSCLC with low tumor mutational burden (TMB). Here, we show that mutations of three chromatin remodeling-related genes, including *KMT2C*, *BCOR* and *KDM5C*, are associated with the ICB response in NSCLC, including NSCLC with low TMB level. Furthermore, this association is further improved by a combined use of *KMT2C/BCOR/KDM5C* mutations with TMB or PD-L1 expression. These data suggest that *KMT2C/BCOR/KDM5C* mutation status has the potential to serve as a predictive biomarker for ICB therapy in NSCLC.

**Abstract:**

Efficient predictive biomarkers are urgently needed to identify non-small cell lung cancer (NSCLC) patients who could benefit from immune checkpoint blockade (ICB) therapy. Since chromatin remodeling is required for DNA repair process, we asked whether mutations in chromatin remodeling genes could increase tumor mutational burden (TMB) and predict response to ICB therapy in NSCLC. Analysis of seven ICB-treated NSCLC cohorts revealed that mutations of three chromatin remodeling-related genes, including *KMT2C*, *BCOR* and *KDM5C*, were significantly associated with ICB response, and combined mutations of these three genes further enhance this association. NSCLC patients with *KMT2C/BCOR/KDM5C* mutations had comparable clinical outcomes to TMB-high patients in terms of objective response rate, durable clinical benefit and overall survival. Although *KMT2C/BCOR/KDM5C* mutations were positively correlated with TMB levels in NSCLC, the association of this mutation with better ICB response was independent of tumor TMB and programmed death-ligand 1 (PD-L1) level, and combination of *KMT2C/BCOR/KDM5C* mutations with TMB or PD-L1 further improve the prediction of ICB response in NSCLC patients. Cancer Genome Atlas (TCGA) pan-cancer analysis suggested that the association of *KMT2C/BCOR/KDM5C* mutations with ICB response observed here might not result from DNA repair defects. In conclusion, our data indicate that *KMT2C/BCOR/KDM5C* mutation has the potential to serve as a predictive biomarker, alone or combined with PD-L1 expression or TMB, for ICB therapy in NSCLC.

## 1. Introduction

Eukaryotic genomes are packaged with histones in chromatin at two different states, loosely and highly compacted states [1,2]. Gene transcription and DNA replication require chromatin to be transformed from a compacted to a loosely wrapped state so that genomic DNA is accessible to transcription and replication machinery. The transformation process between these two chromatin states is called chromatin remodeling [1,2]. Functionally, proteins involved in chromatin remolding can be classified into three groups called writer, eraser and reader. Proteins, including histone methyltransferases, that mediate the addition of epigenetic marks onto histone are called writer, whereas proteins like histone demethylases that remove these marks are called eraser. Readers are proteins that recognize and dock onto those epigenetic marks, exerting a delicate control of reversible DNA packing and unpacking [1,2].

Numerous studies have demonstrated that mis-writing, mis-reading or mis-erasing of the epigenetic marks in chromatin is a common event in a wide range of human cancers, contributing to oncogenesis through mechanisms including activation oncogenic genomic loci that are repressed in the normal physiologic state or silence tumor suppressor genes that utilize chromatin remodeling as part of its normal functions [2,3]. Gene mutation is probably the main cause of the above dysfunction of chromatin remodeling. Recurrent somatic mutations in chromatin remodeling-related genes have been detected with high frequency in human cancers, and three of them, including *KMT2D*, *ARID1A* and *KMT2C*, were the third, fifth and seventh-most commonly mutated cancer genes in a pan-cancer cohort containing around 33,000 cases [4,5].

Lung cancer is the leading cause of cancer deaths worldwide [6]. Approximately 80% of lung cancers are non-small cell lung cancer (NSCLC), of which lung adenocarcinoma (LUAD) and lung squamous cell carcinoma (LUSC) are the two major histologic subtypes [7]. Immune checkpoint blockade (ICB) therapy, which enhances antitumor immune response by using antibodies blocking inhibitory immune-checkpoint proteins such as programmed cell death protein 1 (PD-1) and its ligand PD-L1, has demonstrated significant clinical benefit for NSCLC [8,9]. PD-L1 expression and tumor mutational burden (TMB) are the two main biomarkers that are currently used to predict ICB effectiveness for NSCLC patient selection in clinical practice [10]. However, a recent study showed that 44–50% of NSCLC patients with high PD-L1 expression or high TMB did not respond to ICB while 12–15% of patients with low PD-L1 expression or low TMB achieved a partial or complete response [11]. Studies also suggested that abnormalities in DNA repair genes predicted ICB response [12,13,14], and the explanation for this is that DNA repair defects cause a higher mutation load in cancer. Since chromatin remodeling is required for DNA repair process, one may assume that mutations in chromatin remodeling genes could also increase TMB in cancer and predict ICB response. In this study, we performed an integrated analysis to test this assumption by incorporating a large amount of clinical and genomic data from NSCLC cohorts and TCGA pan-cancer cohorts.

## 2. Methods

### 2.1. In-House NSCLC Cohort

A total of 38 NSCLC patients with clinical response data to anti-PD-1 antibodies were analyzed [15] (Table S1). Genetic alterations in tumors were identified using FoundationOne next-generation sequencing (NGS) assay (Cambridge, MA, USA) as we described previously [15]. Genomic variants were filtered against common single-nucleotide polymorphisms found in dbSNP (www.ncbi.nlm.nih.gov/snp/, accessed on 27 January 2022) to eliminate germline polymorphisms [16]. Clinical response was assessed using Response Evaluation Criteria in Solid Tumors version 1.1 criteria. Objective response rate (ORR) was defined as the percentage of patients with complete or partial response after treatment. Durable clinical benefit (DCB) was defined as longer than 6 months with no progressive disease [17]. This study was approved by the local ethics committee (CHUN, IE-2017-905). Written informed consent was obtained from all participants.

### 2.2. Publicly Available NSCLC Cohorts

By searching the literature, we identified five NSCLC immunotherapy cohorts and used them as discovery sets here. These cohorts includes Rizvi 2015 [18], Hellmann 2018 [19], Miao 2018 [20], Rizvi 2018 [17] and Samstein 2019 [21] that contain tumor exome sequencing data. The patients in these cohorts were treated with anti-PD-1/PD-L1 agents alone or with combination of anti-CTLA-4 antibodies. Since most of the samples in Rizvi 2015 cohort were overlapped with that in Miao 2018 cohort, the non-overlapped samples in the former cohort were merged into the latter one. All these cohorts were annotated with both ICB response and patient’s progression-free survival information, except that Samstein 2019 cohort only with overall survival information. Notably, cohorts Rizvi 2018 and Samstein 2019 have overlapped samples. Since Samstein 2019 cohort was only used here to test the associations of risk factors with patient’s’ overall survival, while Rizvi 2018 cohort was used to test correlations with ICB response and patient’s progression-free survival, we did not remove the overlapped samples from Samstein 2019 cohort. Genomic and clinical data for these cohorts were retrieved from cBioPortal portal (https://www.cbioportal.org, accessed on 3 June 2021).

Data from circulating cell-free tumor DNA (ctDNA)-based NGS carried out in patients enrolled in the randomized phase II/III POPLAR/OAK trials [22], which compared atezolizumab versus docetaxel as second-line treatment in patients with NSCLC, was used as a validation cohort here. The sequencing data and patient clinical information for this cohort were obtained from the original study [22]. The EGFR-positive and ALK-positive samples were excluded as suggested [22]. To minimize the false-negative rates for gene mutation, only samples with median_exon_coverage ≥2000 were included in our study.

To assess whether the survival benefit from *KMT2C/BCOR/KDM5C* mutations were specific for NSCLC patients treated with ICB, three additional NSCLC cohorts were analyzed in this study, including a non-ICI-treated cohort from Zehir et al. study [23], and the LUAD and LUSC cohorts from Cancer Genome Atlas (TCGA) portal.

ORR and DCB definitions for external cohorts were same as we used for the in-house cohort. The baseline features of NSCLC in the above cohorts are presented in Table S1.

### 2.3. TCGA Pan-Cancer Cohorts

Besides TCGA LUAD and LUSC cohorts, we also tested another 31 TCGA cohorts to unravel the potential biological effects of *KMT2C*, *BCOR* and *KDM5C* mutations. Notably, results related to *KMT2C*, *BCOR* and *KDM5C* mutations were not available for 12 of the 32 TCGA cohorts because those cohorts don’t contain sequencing data for all these three genes or have less than four samples carrying mutations in any of the three genes. The mutation annotation format (MAF) files and upper-quartile normalized RNA-Seq data for these TCGA cohorts were downloaded from the GDC data portal (https://portal.gdc.cancer.gov, accessed on 9 September 2021) and Firehose (http://gdac.broadinstitute.org/, accessed on 12 September 2021), respectively. The RNA-Seq expression data were further log2 transformed by using the Voom algorithm implemented in R package limma. The patients’ survival information was retrieved from a manually curated file [24]. The TCGA study abbreviations were described in the Supplementary Methods.

The following are TCGA study abbreviations. ACC: adrenocortical carcinoma; BLCA: bladder urothelial carcinoma; BRCA: breast invasive carcinoma; CESC: cervical squamous cell carcinoma and endocervical adenocarcinoma; CHOL: cholangiocarcinoma; COAD: colon adenocarcinoma; DLBC: lymphoid neoplasm diffuse large b-cell lymphoma; ESCA: esophageal carcinoma; GBM: glioblastoma multiforme; HNSC: head and neck squamous cell carcinoma; KICH: kidney chromophobe; KIRC: kidney renal clear cell carcinoma; KIRP: kidney renal papillary cell carcinoma; LAML: acute myeloid leukemia; LCML: Chronic Myelogenous Leukemia; LGG: brain lower grade glioma; LIHC: liver hepatocellular carcinoma; LUAD: lung adenocarcinoma; LUSC: lung squamous cell carcinoma; MESO: mesothelioma; OV: ovarian serous cystadenocarcinoma; PAAD: pancreatic adenocarcinoma; PCPG: pheochromocytoma and paraganglioma; PRAD: prostate adenocarcinoma; READ: rectum adenocarcinoma; SARC: sarcoma; SKCM: skin cutaneous melanoma; STAD: stomach adenocarcinoma; TGCT: testicular germ cell tumors; THCA: thyroid carcinoma; THYM: thymoma; UCEC: uterine corpus endometrial carcinoma; UCS: uterine carcinosarcoma; UVM: uveal melanoma.

### 2.4. Mutation Data Analysis and TMB Calculation

MAF files were read and analyzed using R package “maftools” to identify and summarize nonsynonymous somatic mutations. According to the instructions of this package, variants including frame shift del, frame shift ins, splice site, translation start site, nonsense mutation, nonstop mutation, in frame del, in frame ins and missense mutation are classified as nonsynonymous mutations [25]. For genomic data from cBioPortal, MAFs annotated with UniProt isoforms were used.

As described previously, TMB was defined as the total number of nonsynonymous mutations per megabase (Mb) of genome examined [16,17]. For whole-exome sequencing data, 38 Mb was adopted as the estimated exome size [16]. For MSK-IMPACT panel-sequenced samples, the exonic coverage sizes used for TMB calculation were set as 0.98, 1.06, and 1.22 Mb in 341-, 410- and 468- gene panels, respectively [17].

### 2.5. Chromatin Remodeling-Related Genes and Definition for KMT2C/BCOR/KDM5C Mutations

Lists of chromatin remodeling-related genes were obtained from our and other previous reports [26,27,28]. *KMT2C/BCOR/KDM5C* mutations were defined as a tumor sample that carries mutation in any one of three genes *KMT2C*, *BCOR* and *KDM5C*.

### 2.6. DNA Repair Pathway Gene Expression Score (RPS)

Three genes including *Rif1*, *XRCC5* and *PARPBP* that antagonize homologous recombination (HR), together with *RAD51* that is upregulated following the presence of HR defects, were used to build gene signature for RPS [29]. The RPS was defined as the sum of expression levels of these four genes multiplied times -1. Z-score transformed mRNA values of each gene were used in RPS calculation as described previously [29].

### 2.7. Intratumoral Immune Cell Composition Analysis

Intratumoral immune cell subtype fractions were calculated using CIBERSORT algorithm (https://cibersort.stanford.edu/, accessed on 29 September 2022) based on normalized gene expression data [30,31]. The LM22 gene signatures (corresponded to 22 sorted immune cell subsets), which were experimentally validated for their prediction accuracy [30], were chosen for CIBERSORT analysis in this study.

### 2.8. Gene Set Enrichment Analysis (GSEA)

GSEA was performed using R clusterProfiler package as we described previously [32]. A collection of Kyoto Encyclopedia of Genes and Genomes (KEGG) and hallmark pathway gene sets (version 7.4) was tested in GSEA analysis [33]. Gene set permutations were performed 1000 times for each analysis. Normalized enrichment score |NES| > 1, nominal BH-adjusted *p*-value < 0.05 and FDR q-value < 0.25 were considered significant gene sets.

### 2.9. Statistics

Statistics were performed using R packages including stats, metafor, survival and survminer. Wilcoxon rank sum test was used for unpaired two-sample comparisons. Chi-squared test was employed for analysis of count variable. Fisher’s exact test was used when the expected frequency for any cell was less than five. Pearson correlation was used to examine the correlation between RPS and TMB levels in TCGA pan-cancer cohorts. Logistic regression was used to calculate odds-ratios (OR) for the associations of *KMT2C/BCOR/KDM5C* mutations with clinical variables. Cox proportional hazards regression and Kaplan-Meier survival curves with log-rank test were used to analyze the association between risk factors and patient’s survival. The overall hazard ratio (HR) of a variable of interest was calculated using a random-effects model. The significance of the overall effects across multiple cohorts was estimated by Z test. All statistical analyses were two-sided and considered significant when *p* < 0.05.

## 3. Results

### 3.1. Mutations of Chromatin Remodeling-Related Genes KMT2C, BCOR and KDM5C Were Significantly Associated with ICB Response in NSCLC

**Figure 1:**
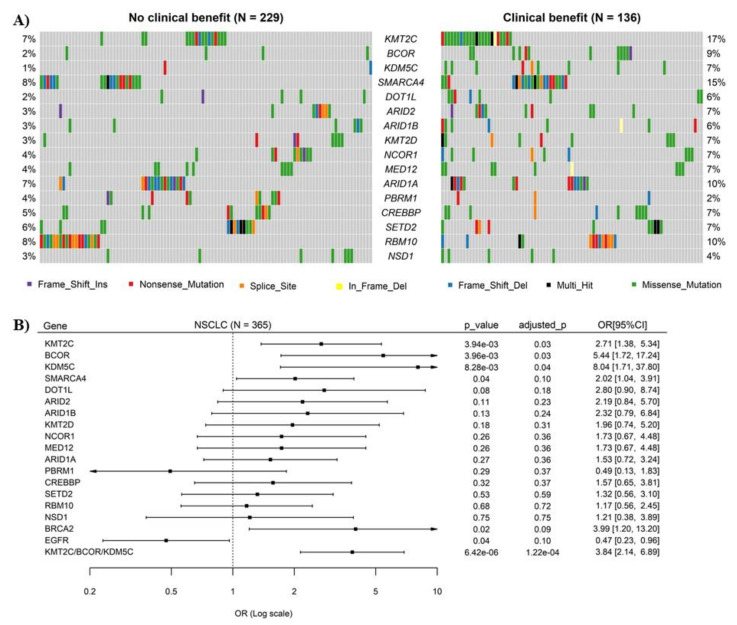
Mutations of chromatin remodeling-related genes and their correlations with response of non-small cell lung cancer (NSCLC) to immune checkpoint blockade (ICB) therapy. (**A**) Oncoplot displaying the somatic landscape of 15 chromatin remodeling-related genes in NSCLC. Each column represents a sample. Samples without mutations in any of the 15 genes were not shown in the figure. The numbers shown at left and right sides represent the gene mutation rates. Variants annotated as Multi_Hit are those genes which are mutated more than once in the same sample. (**B**) Logistic regression analysis of the correlation of mutations of 15 chromatin remodeling-related genes with ICB response in NSCLC. The ORs are shown in forest plots, in which the squares and horizontal lines represent the OR and 95% CI for the corresponding gene. Mutations of EGFR and BRCA2 were used here as positive controls. *p*-values were adjusted using Benjamini & Hochberg’s method.

Three NSCLC cohorts were analyzed as discovery sets here. Sequencing data for 136 genes were available in all three cohorts, and 16 of them were chromatin remodeling-related genes. No mutually exclusive mutation patterns were observed among these genes (Figure S1). All 16 genes, except *PBRM1*, had relatively higher mutation rates in patients with DCB than those with no durable benefit (NDB) (Figure 1A), and logistic regression analysis showed that mutations of three genes, including *KMT2C*, *BCOR* and *KDM5C*, were significantly associated with ICB response (Figure 1B). Overall, the somatic mutations of these three genes were evenly distributed along their coding sequences and no difference in distribution pattern was observed between DCB and NDB samples (Figure S2). No significant correlations of mutation rates of these 3 genes with patient’s age, gender and tumor stages were observed (Table S2).

### 3.2. Combination of KMT2C, BCOR and KDM5C Mutations Improved the Prediction of ICB Response in NSCLC

**Figure 2:**
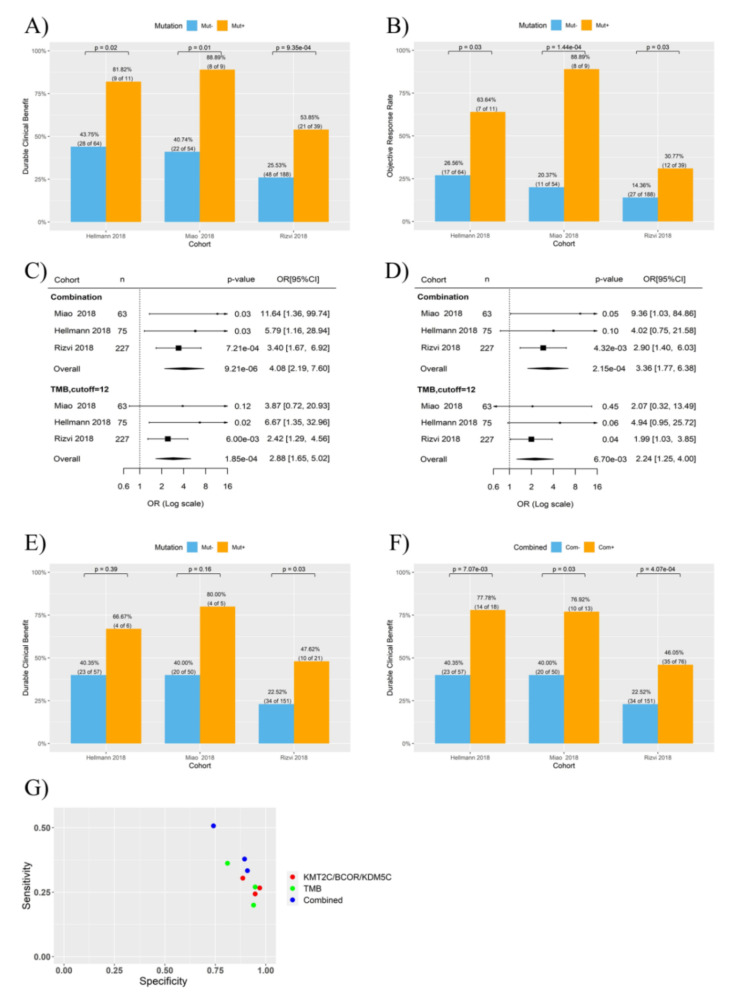
Association of *KMT2C/BCOR/KDM5C* mutations with NSCLC response to ICB therapy. Three ICB therapy cohorts were tested as indicated. TMB-high and TMB-low subgroups were stratified by using 12 mutations/Mb as cutoff. (**A**,**B**) Histogram showing the DCB (**A**) or ORR (**B**) rates of NSCLC patients with or without *KMT2C/BCOR/KDM5C* mutations. (**C**,**D**) Univariate (**C**) and multivariate (**D**) logistic regression analysis of the correlations of ICB response with TMB level and *KMT2C/BCOR/KDM5C* mutations. Both *KMT2C/BCOR/KDM5C* mutations (indicated as combination in the plot) and TMB were used as dichotomous variables in regression analysis. (**E**) Histogram showing the DCB rates of low-TMB patients with or without *KMT2C/BCOR/KDM5C* mutations. (**F**) Histogram showing the DCB rates of two groups of NSCLC stratification based on combination of TMB and *KMT2C/BCOR/KDM5C* mutations. Com-: NSCLC with neither high TMB nor *KMT2C/BCOR/KDM5C* mutations. Com+: NSCLC with either high TMB or *KMT2C/BCOR/KDM5C* mutations. (**G**) Specificity and sensitivity for prediction of DCB in NSCLC. Each dot represents one cohort. *KMT2C/BCOR/KDM5C* mutations, TMB, and combination of *KMT2C/BCOR/KDM5C* mutations were used as predictors as indicated.

Interestingly, a combination of mutations in *KMT2C*, *BCOR* and *KDM5C* (i.e., NSCLC with mutation in any one of the three genes regarded as *KMT2C/BCOR/KDM5C* mutation positive) further improve the efficiency on ICB response prediction (OR 3.84, 95% CI 2.14∓6.89, *p* = 1.16 × 10^−4^) (Figure 1B). Analysis on individual cohorts showed that DCB rates were 81.82%, 88.89% and 53.85% for NSCLC with *KMT2C/BCOR/KDM5C* mutations in the three cohorts, respectively, which are significantly higher than that in NSCLC with wild-type *KMT2C/BCOR/KDM5C* (Figure 2A). Similar significant association was found when ORR was tested in the three cohorts (Figure 2B).

Previously, we and others established cutoff values of 9-18 mutations/Mb to determine NSCLC patients with high TMB [15,21,34,35]. We found here that TMB-high and TMB-low subgroups stratified using 12 mutations/Mb as cutoff had the most difference in DCB rate between the two subgroups (Figure S3). Logistic regression indicated that both TMB and *KMT2C/BCOR/KDM5C* mutations were associated with DCB rate in the three cohorts (*p* < 0.001) (Figure 2C), and the association of *KMT2C/BCOR/KDM5C* mutations with DCB remained significant after adjusted for TMB level (Figure 2D). Interestingly, association of TMB with DCB was weakened by adjustment for *KMT2C/BCOR/KDM5C* mutations (Figure 2D).

### 3.3. KMT2C/BCOR/KDM5C Mutations Combined with TMB and PD-L1 Level Further Improved the Prediction of ICB Response in NSCLC

*KMT2C/BCOR/KDM5C* mutations predicted ICB response even in NSCLC with low TMB (Figure 2E). Moreover, higher statistical significance and prediction sensitivity (true positive rate) were achieved when patients were stratified based on a combination of TMB and *KMT2C/BCOR/KDM5C* mutations (i.e., patients with high TMB or *KMT2C/BCOR/KDM5C* mutations as ‘potentially responsive’ group and the rest as ‘non-responsive group) (Figure 2F,G). This is because more patients who did benefit from ICB treatment were classified into ‘potentially responsive’ group, although the DCB rate of this group was not increased (Figure 2F).

Some of the samples in the Hellmann 2018 and Rizvi 2018 cohorts were annotated with PD-L1 expression data. Analysis of these samples showed that PD-L1 expression (tumors with >30% positive cells as cut-off) was strongly correlated with DCB in both cohorts (Figure S4A–C). The association of *KMT2C/BCOR/KDM5C* mutations with DCB was not affected in multivariate cox regression after adjusted for PD-L1 expression (Figure S4D–E). Notably, when patients were stratified based on the combination of PD-L1 expression and *KMT2C/BCOR/KDM5C* mutation status, DCB rate difference was more significant between two patient subgroups (i.e., patients with *KMT2C/BCOR/KDM5C* mutations and/or positive PD-L1 vs. negative for both the mutation and PD-L1 expression) (Figure S4F,G). A combined usage of TMB, PD-L1 expression and *KMT2C/BCOR/KDM5C* mutations further enhanced the significance in DCB difference between patient subgroups (Figure S4H). Notably, whole tumor tissues were used for gene expression measurement in these two cohorts, and the detected PD-L1 may have derived from both tumor and tumor-infiltrated immune cells; therefore, further studies are needed to support the findings shown in Figure S4.

### 3.4. KMT2C/BCOR/KDM5C Mutations Were Associated with Survival of NSCLC Patients Treated with ICB

**Figure 3:**
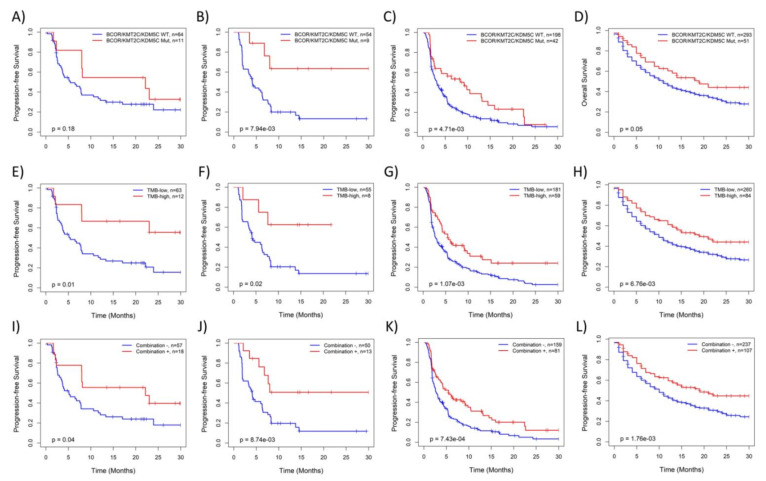
Kaplan-Meier analysis of the associations of NSCLC survival with *KMT2C/BCOR/KDM5C* mutations and TMB level with: (**A**–**D**) patient stratified based on *KMT2C/BCOR/KDM5C* mutations, (**E**–**H**) patient stratified based on TMB level (12 mutations/Mb as cutoff), (**I**–**L**) patient stratified based on a combination of *KMT2C/BCOR/KDM5C* mutations and TMB level. Four ICB treated cohorts were analyzed, including Hellmann 2018 (**A**,**E**,**I**), Miao 2018 (**B**,**F**,**J**), Rizvi 2018 (**C**,**G**,**K**) and Samstein 2019 cohorts (**D,H,L**). PFS time was used in survival analysis except for Samstein 2019 in which only overall survival (OS) follow-up data is available.

Kaplan-Meier analysis further confirmed that NSCLCs with *KMT2C/BCOR/KDM5C* mutations (Figure 3A–D) or high TMB (Figure 3E–H) had significantly better survival than those with wild-type *KMT2C/BCOR/KDM5C* or low TMB. Similar to the data shown in Figure 2F, association of patient survival with combined *KMT2C/BCOR/KDM5C*-mutation/TMB was more significant in statistics due to the increased size of ‘potentially responsive’ group, although the survival difference between the two groups of patients did not increase (Figure 3I–L). Additionally, *KMT2C/BCOR/KDM5C* mutation-related survival benefit was not found in NSCLC from a non-ICB treated cohort and TCGA cohorts (Figure S5), indicating that prognostic power of *KMT2C/BCOR/KDM5C* mutations is restricted to NSCLC patients treated with ICB.

### 3.5. Validation of the Association between KMT2C/BCOR/KDM5C Mutations and ICB Response in In-House Cohort and ctDNA-Based NGS Cohort

**Figure 4:**
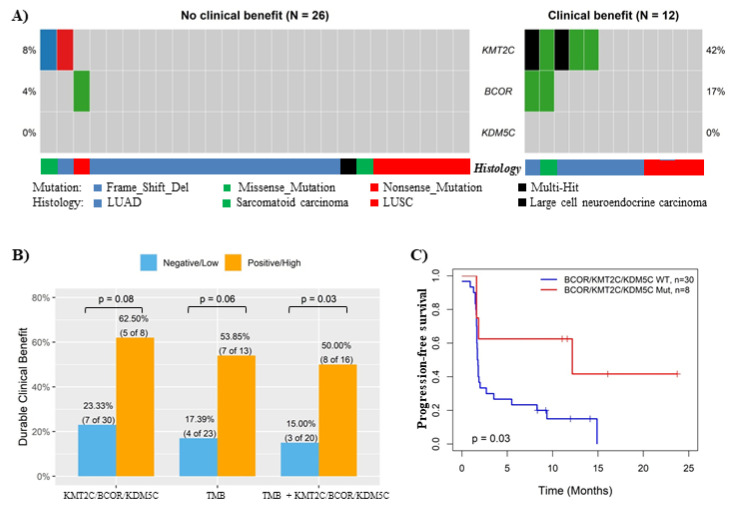
Validation of the association between *KMT2C/BCOR/KDM5C* mutations and ICB response in in-house cohort. (**A**) Oncoplot displaying the somatic mutations of KMT2C, BCOR and KDM5C in in-house NSCLC cohort. Each column represents a sample. The numbers shown at left and right sides represent the gene mutation rates. (**B**) Histogram showing the DCB rates of NSCLC patient subgroups stratified based on *KMT2C/BCOR/KDM5C* mutation status, TMB level (cutoff: 16 mutations/Mb) or combined TMB & *KMT2C/BCOR/KDM5C* mutations as indicated. Combination negative: NSCLC with neither high TMB nor *KMT2C/BCOR/KDM5C* mutations. Combination positive: NSCLC with either high TMB or *KMT2C/BCOR/KDM5C* mutations. (**C**) Kaplan-Meier analysis of the associations of NSCLC survival with *KMT2C/BCOR/KDM5C* mutations.

In the in-house NSCLC cohort, *KMT2C* and *BCOR* also showed much higher mutation rates in patients with DCB than those with NCB (no *KDM5C* mutation was identified in both subgroups) (Figure 4A), and the DCB rate was 2.68 fold (*p* = 0.08) higher in NSCLC carrying *KMT2C/BCOR/KDM5C* mutations than wild-type tumors (Figure 4B). Combined use of *KMT2C/BCOR/KDM5C* mutations and TMB further increased predictive efficiency on ICB response in this cohort (*p* = 0.03) (Figure 4B), which was consistent with the results obtained from other cohorts (Figure 2). NSCLC with *KMT2C/BCOR/KDM5C* mutations also had better progression-free survival than those without such mutations (*p* = 0.03) (Figure 4C).

**Figure 5:**
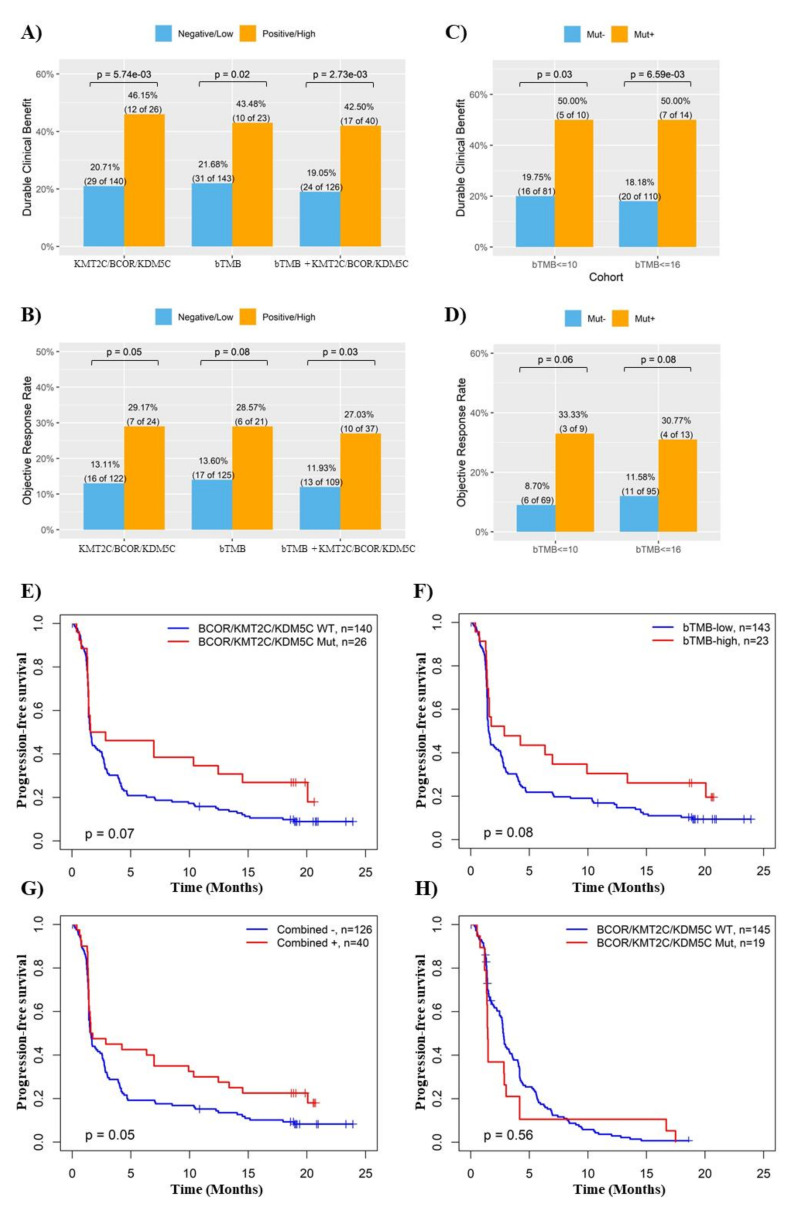
Validation of the association between *KMT2C/BCOR/KDM5C* mutations and ICB response in ctDNA-based NGS cohort. (**A**,**B**) Histogram showing the DCB (**A**) or ORR (**B**) rates of NSCLC patient subgroups stratified based on *KMT2C/BCOR/KDM5C* mutation status, bTMB level or combined bTMB & *KMT2C/BCOR/KDM5C* mutations as indicated. We used 23 mutations/Mb as cutoff for high bTMB level. This cutoff was used here based on the data from Supplementary Figure S7. Combination negative: NSCLC with neither high TMB nor *KMT2C/BCOR/KDM5C* mutations. Combination positive: NSCLC with either high TMB or *KMT2C/BCOR/KDM5C* mutations. (**C**,**D**) Histogram showing the DCB (**C**) or ORR (**D**) rates of low-bTMB patients with or without *KMT2C/BCOR/KDM5C* mutations. (**E**–**G**) Kaplan-Meier analysis of the associations of NSCLC survival with *KMT2C/BCOR/KDM5C* mutations (**E**), bTMB level (**F**), or a combination of *KMT2C/BCOR/KDM5C* mutations and bTMB level (**G**). (**H**) Associations of patient survival with *KMT2C/BCOR/KDM5C* mutations in NSCLC treated with docetaxel.

Recently, ctDNA-based NGS analysis indicated that NSCLC patients with high blood TMB (bTMB) receiving anti-PD-L1 antibody atezolizumab on the OAK/POPLAR trials had improved survival compared with bTMB-low patients [22]. Re-analysis of this cohort indicated that like bTMB, *KMT2C/BCOR/KDM5C* mutations in ctDNA was also a significant favorable factor for ICB response in NSCLC (*p* < 0.01 and 0.05 for DCB and ORR rates, respectively) (Figures S6 and S7 and Figure 5A,B). The associations of *KMT2C/BCOR/KDM5C* mutations with DCB and ORR remained significant even in NSCLC patients with low bTMB (Figure 5C,D). Moreover, improved prediction for ICB response and progression-free survival by combination of *KMT2C/BCOR/KDM5C* mutations with bTMB were also observed in the OAK/POPLAR cohort (Figure 5A,B,E–G). Notably, survival benefit from *KMT2C/BCOR/KDM5C* mutations was only observed in patients treated with atezolizumab but not docetaxel (Figure 5H). Therefore, it was not surprising to find that atezolizumab had better therapeutic effects over docetaxel only in NSCLC with *KMT2C/BCOR/KDM5C* mutations (Figure S8).

### 3.6. KMT2C/BCOR/KDM5C Mutations Were Associated with Increased TMB and Immunogenicity in NSCLC

**Figure 6:**
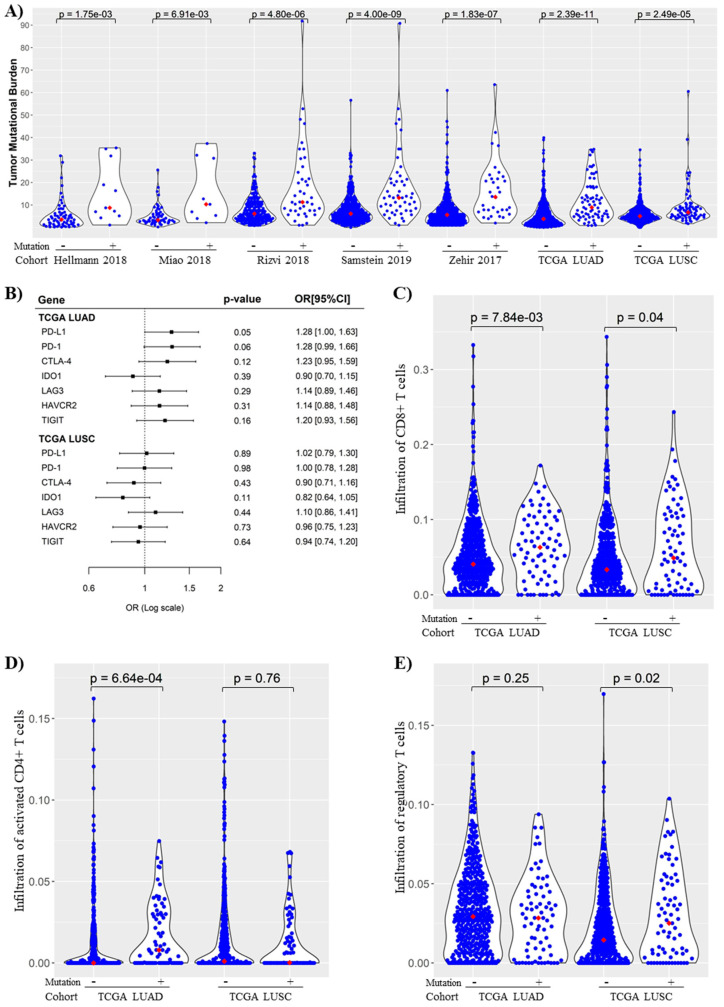
*KMT2C/BCOR/KDM5C* mutations were associated with increased tumor mutational burden (TMB) and immunogenicity in NSCLC. (**A**) Correlation between *KMT2C/BCOR/KDM5C* mutations and TMB levels in NSCLC. Seven NSCLC cohorts were analyzed as indicated. Each dot represents one sample and red dots represent median TMB values. (**B**) Logistic regression analysis of the correlation of *KMT2C/BCOR/KDM5C* mutations with expression of immune checkpoint-related genes in TCGA LUAD and LISC cohorts (mutant as event and wild-type as non-event). Univariate logistic regression was used to compute the OR (per one-SD) with the increase of gene expression (used as a continuous variable). (**C**–**E**) Correlation of *KMT2C/BCOR/KDM5C* mutations with infiltration of CD8^+^ T cells (**C**), activated CD4^+^ T cells (**D**) and Regulatory T cells (**E**) in TCGA LUAD and LISC cohorts. Each dot represents one sample and red dots represent median values of cell infiltration level. Wilcoxon signed rank test was used to compare the difference in *KMT2C/BCOR/KDM5C* mutant and wild-type groups.

To unravel potential molecular mechanisms for the prognostic capability of *KMT2C/BCOR/KDM5C* mutations, we analyzed the association of the mutations with immunogenicity in NSCLC. Tumors with *KMT2C/BCOR/KDM5C* mutations had significantly higher TMB levels than those with wild-type alleles in all seven NSCLC cohorts (Figure 6A). Among seven immune checkpoint-related genes tested, *KMT2C/BCOR/KDM5C* mutations were also positively correlated with expression of PD-L1 and PD-1 in NSCLC (Figure 6B). Fraction analysis of 22 immune cell subsets in TCGA NSCLC cohorts revealed that infiltration levels of three subsets were significantly different between *KMT2C/BCOR/KDM5C* wild-type and mutant groups in TCGA LUAD and LUSC cohorts (Figure S9). Among these three subsets, infiltration of CD8^+^ or activated CD4^+^ T cells was increased while infiltration of regulatory T cells was decreased in NSCLC with *KMT2C/BCOR/KDM5C* mutations (Figure 6C–E).

### 3.7. Association of KMT2C/BCOR/KDM5C Mutations with DNA Repair Pathway Score in NSCLC

Analysis of 33 TCGA pan-cancer cohorts revealed that DNA repair pathway score was negatively correlated with TMB in most cancer types, particularly LUAD (Figures S10 and S11). Since chromatin remodeling is a necessary step in the DNA repair process, one may assume that *KMT2C/BCOR/KDM5C* mutations induce increase of TMB and immunogenicity through disrupting DNA repair. However, analysis of TCGA NSCLC cohorts showed no significant correlation of DNA repair pathways with *KMT2C/BCOR/KDM5C* mutations, while the score was significantly associated with *TP53* mutation (Figure S12A). Moreover, analysis of TCGA pan-cancer transcriptional and mutational data revealed that the association of *KMT2C/BCOR/KDM5C* mutations with DNA repair pathway score did not well correlate with the association of *KMT2C/BCOR/KDM5C* mutations with TMB in the same cancer type (Figure S12B,C).

### 3.8. Hallmark and KEGG Pathway Gene Sets Analysis of NSCLC with or without KMT2C/BCOR/KDM5C Mutation

The loose connection between *KMT2C/BCOR/KDM5C* mutations with DNA repair defects (Figures S10–S12) suggested that other mechanisms may mediate the association of this 3-gene mutation with ICB response. To unravel such potential mechanisms, GSEA analysis was performed in TCGA pan-cancer cohorts. In TCGA NSCLC cohorts, a total of 35 pathway gene sets were found to be significantly suppressed or activated in tumors with *KMT2C/BCOR/KDM5C* mutations (Table S3). Ten of the gene sets, most of which are related to metabolisms such as arachidonic acid metabolism, were suppressed by *KMT2C/BCOR/KDM5C* mutations in both LUAD and LUSC cohorts (Figure S13A). Gene sets affected by *KMT2C/BCOR/KDM5C* mutations in LUAD but not LUSC were mainly related to cell cycle regulation (Figure S13B,C), while gene sets affected by the mutations in LUSC but not LUAD were mainly related to inflammatory response (Figure S13D,E).

## 4. Discussion

Extensive efforts have been made recently to develop predictive markers for identification NSCLC patients who could benefit from ICB therapy. In this study, we found that mutations of three chromatin remolding-related genes, including *KMT2C*, *BCOR* and *KDM5C*, predicted ICB response in NSCLC.

*KMT2C* is a histone methyltransferase that methylates ‘Lys-4’ of histone H3 [5], while *KDM5C* is a histone demethylase that specifically demethylates ‘Lys-4’ of histone H3 [36]. *BCOR* takes part in the polycomb repressive complex (PRC) 1.1, which catalyzes the ubiquitination of Lys119 on histone H2A [37]. Therefore, *KMT2C* and *BCOR* function as ‘writers’ while *KDM5C* as an ‘eraser’ in chromatin remodeling. Since chromatin remodeling is a necessary step in DNA repair process, we initially thought that that the positive association of *KMT2C/BCOR/KDM5C* mutations with increased immunogenicity in NSCLC could be attributed to DNA repair defects and increased mutation rates. However, pairwise correlations among *KMT2C/BCOR/KDM5C* mutations, TMB and DNA repair pathway score do not support this assumption (Figures S10–S12).

GSEA analysis showed that nearly all the gene sets suppressed by *KMT2C/BCOR/KDM5C* mutations in both LUAD and LUSC were related to cell metabolism. This is not surprising since chromatin remodeling is intimately tied to metabolic processes, and many intermediary metabolites are required co-factors for histone post-translational modification [38]. Additionally, due to dysregulated metabolic activity in tumor cells, conditions in the tumor microenvironment will typically impose metabolic stress on infiltrating immune cells that may lead to impaired antitumor immune responses [39]. These data suggested that chromatin remodeling and tumor immunogenicity could be correlated with each other at the metabolism level. Cyclooxygenase 2 (COX2) is overexpressed in numerous cancers including NSCLC and functions as an immunosuppressor through arachidonic acid-derived Prostaglandin E_2_ [40]. Our data showed that *KMT2C/BCOR/KDM5C* mutations were correlated with suppression of arachidonic acid metabolism gene set in both LUAD and LUSC, providing another potential mechanism for the association between the mutation and increased immunogenicity in NSCLC.

Studies have demonstrated that several chromatin remolding genes such as *SNF5* and *CHD5* might possess specialized roles in addition to their participation in chromatin remolding [41,42]. The same may go for *KMT2C*, *BCOR* and *KDM5C*. KMT2C was reported to directly interact with TP53 and be required for activation of TP53 target genes, and loss of KMT2C in cancer may contribute to a more stem-cell like state and mesenchymal phenotype [5]. BCOR binds to proto-oncogene BCL-6 and enhances BCL-6-mediated transcriptional repression. Functional studies suggested important roles of this gene in pluripotency maintenance and cell fate determination [37]. Further studies are mandatory to clarify whether these functions are relevant to the prediction power of *KMT2C/BCOR/KDM5C* mutations for and response of NSCLC to ICB therapy.

Since exome sequencing covered less than 500 genes in most of the ICB-treated tested in this study, the mutation data of most chromatin remodeling-related genes were not available for these samples. It is unclear whether mutations in chromatin remodeling-related genes other than *KMT2C*, *BCOR* and *KDM5C* are associated with ICB response in NSCLC. Notably, it was previously reported that mutation in *PBRM1*, a component of chromatin remodeling complex SWI/SNF-B, was associated with clinical benefit from ICB therapy for clear cell renal cell carcinoma [20,43]. In this study, however, such association was not observed in NSCLC.

One limitation of this study is that *KMT2C/BCOR/KDM5C* mutation rate is relatively low in NSCLC (around 15%), which means that if this mutation status was used as biomarker for patient selection in routine clinical practice, many patients who will benefit from ICB treatment may not be chosen for this therapy. Our findings indicated that this low sensitivity issue could be overcome by combined use of PD-L1 or TMB markers, since data from multiple cohorts showed that classifying NSCLC patients with either positive PD-L1, high-TMB or *KMT2C/BCOR/KDM5C* mutations into ‘potentially responsive’ could largely increase the sensitivity of ICB-response prediction without hurting predictive specificity (Figure 2F, Figure 4B, Figure 5A,B). From this point of view, NSCLC with negative PD-L1, low-TMB and wild-type *KMT2C/BCOR/KDM5C* is unlikely to be responsive to ICB therapy, and treatments other than ICB may be considered for those NSCLC patients.

## 5. Conclusions

In summary, our data indicate that tumor *KMT2C/BCOR/KDM5C* mutation status, alone or combined with PD-L1expression or TMB, is a promising predictor for response of NSCLC to ICB therapy. Additionally, excellent association of this mutation status in plasma with ICB response supports it can potentially serve as a non-invasive biomarker for ICB therapy.

## Data Availability

The sequencing data for the in-house NSCLC cohort and the processed bioinformatics data are available from the corresponding author upon request. The other NGS cohorts analyzed in this study are publicly available from online databases.

## Supplementary Materials

The following are available online at www.mdpi.com/article/10.3390/cancers14112816/s1, Table S1. Demographic features of patients in seven NSCLC cohorts, Table S2. Mutation rates of KMT2C, BCOR and KDM5C in NSCLC subgroups, Table S3. Hallmark and KEGG pathway gene sets that are suppressed or activated by BCOR/KMT2C/KDM5C mutations, Figure S1. Heatmap displaying consistency and exclusivity correlations among 15 chromatin remodeling-related genes in NSCLC, Figure S2. Lollipop plot displaying mutation distribution and protein domains for three chromatin remodeling-related genes in NSCLC, Figure S3. Comparison of DCB rates between NSCLC patients with low and high tumor mutation burden (TMB) levels., Figure S4. Association of PD-L1 expression, alone or in combination, with NSCLC response to ICB therapy, Figure S5. Kaplan-Meier analysis of the associations of *KMT2C/BCOR/KDM5C* mutations with prognosis of NSCLC without ICB therapy record. Figure S6. Oncoplot displaying the somatic mutations of KMT2C, BCOR and KDM5C in NSCLC of OAK/POPLAR cohort. Figure S7. Comparison of ICB response between NSCLC patients with low and high blood TMB (bTMB) levels. Figure S8. Comparison of the therapeutic effects of atezolizumab and docetaxel in different NSCLC subgroups of OAK/POPLAR cohort. Figure S9. Associations of *KMT2C/BCOR/KDM5C* mutations with immune cell subsets infiltration in NSCLC. Figure S10. Correlation between DNA repair pathway score and TMB level in TCGA NSCLC cohorts. Figure S11. Correlation between DNA repair pathway score and TMB level in TCGA pan-cancer cohorts. Figure S12. Association of *KMT2C/BCOR/KDM5C* mutations with DNA repair pathway score in NSCLC. Figure S13. *KMT2C/BCOR/KDM5C* mutations and enrichment of Hallmark and KEGG pathway gene sets.

## References

[1] Kaur J., Daoud A., Eblen S.T. (2019). Targeting Chromatin Remodeling for Cancer Therapy. Curr. Mol. Pharmacol..

[2] Zhao S., Allis C.D., Wang G.G. (2021). The Language of Chromatin Modification in Human Cancers. Nat. Rev. Cancer.

[3] Nair S.S., Kumar R. (2012). Chromatin Remodeling in Cancer: A Gateway to Regulate Gene Transcription. Mol. Oncol..

[4] Kandoth C., McLellan M.D., Vandin F., Ye K., Niu B., Lu C., Xie M., Zhang Q., McMichael J.F., Wyczalkowski M.A. (2013). Mutational Landscape and Significance across 12 Major Cancer Types. Nature.

[5] Fagan R.J., Dingwall A.K. (2019). COMPASS Ascending: Emerging Clues Regarding the Roles of MLL3/KMT2C and MLL2/KMT2D Proteins in Cancer. Cancer Lett..

[6] Bray F., Ferlay J., Soerjomataram I., Siegel R.L., Torre L.A., Jemal A. (2018). Global Cancer Statistics 2018: GLOBOCAN Estimates of Incidence and Mortality Worldwide for 36 Cancers in 185 Countries. CA A Cancer J. Clin..

[7] Breathnach O.S., Freidlin B., Conley B., Green M.R., Johnson D.H., Gandara D.R., O’Connell M., Shepherd F.A., Johnson B.E. (2001). Twenty-Two Years of Phase III Trials for Patients with Advanced Non-Small-Cell Lung Cancer: Sobering Results. J. Clin. Oncol..

[8] Borghaei H., Paz-Ares L., Horn L., Spigel D.R., Steins M., Ready N.E., Chow L.Q., Vokes E.E., Felip E., Holgado E. (2015). Nivolumab versus Docetaxel in Advanced Nonsquamous Non-Small-Cell Lung Cancer. N. Engl. J. Med..

[9] Reck M., Rodríguez-Abreu D., Robinson A.G., Hui R., Csőszi T., Fülöp A., Gottfried M., Peled N., Tafreshi A., Cuffe S. (2016). Pembrolizumab versus Chemotherapy for PD-L1-Positive Non-Small-Cell Lung Cancer. N. Engl. J. Med..

[10] Ilie M., Benzaquen J., Hofman V., Lassalle S., Yazbeck N., Leroy S., Heeke S., Bence C., Mograbi B., Glaichenhaus N. (2017). Immunotherapy in Non-Small Cell Lung Cancer: Biological Principles and Future Opportunities. Curr. Mol. Med..

[11] Ready N., Hellmann M.D., Awad M.M., Otterson G.A., Gutierrez M., Gainor J.F., Borghaei H., Jolivet J., Horn L., Mates M. (2019). First-Line Nivolumab Plus Ipilimumab in Advanced Non-Small-Cell Lung Cancer (CheckMate 568): Outcomes by Programmed Death Ligand 1 and Tumor Mutational Burden as Biomarkers. J. Clin. Oncol..

[12] Le D.T., Durham J.N., Smith K.N., Wang H., Bartlett B.R., Aulakh L.K., Lu S., Kemberling H., Wilt C., Luber B.S. (2017). Mismatch Repair Deficiency Predicts Response of Solid Tumors to PD-1 Blockade. Science.

[13] Karzai F., VanderWeele D., Madan R.A., Owens H., Cordes L.M., Hankin A., Couvillon A., Nichols E., Bilusic M., Beshiri M.L. (2018). Activity of Durvalumab plus Olaparib in Metastatic Castration-Resistant Prostate Cancer in Men with and without DNA Damage Repair Mutations. J. Immunother Cancer.

[14] Samstein R.M., Krishna C., Ma X., Pei X., Lee K.-W., Makarov V., Kuo F., Chung J., Srivastava R.M., Purohit T.A. (2021). Mutations in BRCA1 and BRCA2 Differentially Affect the Tumor Microenvironment and Response to Checkpoint Blockade Immunotherapy. Nat. Cancer.

[15] Heeke S., Benzaquen J., Long-Mira E., Audelan B., Lespinet V., Bordone O., Lalvée S., Zahaf K., Poudenx M., Humbert O. (2019). In-House Implementation of Tumor Mutational Burden Testing to Predict Durable Clinical Benefit in Non-Small Cell Lung Cancer and Melanoma Patients. Cancers.

[16] Chalmers Z.R., Connelly C.F., Fabrizio D., Gay L., Ali S.M., Ennis R., Schrock A., Campbell B., Shlien A., Chmielecki J. (2017). Analysis of 100,000 Human Cancer Genomes Reveals the Landscape of Tumor Mutational Burden. Genome Med..

[17] Rizvi H., Sanchez-Vega F., La K., Chatila W., Jonsson P., Halpenny D., Plodkowski A., Long N., Sauter J.L., Rekhtman N. (2018). Molecular Determinants of Response to Anti–Programmed Cell Death (PD)-1 and Anti–Programmed Death-Ligand 1 (PD-L1) Blockade in Patients With Non–Small-Cell Lung Cancer Profiled With Targeted Next-Generation Sequencing. J. Clin. Oncol..

[18] Rizvi N.A., Hellmann M.D., Snyder A., Kvistborg P., Makarov V., Havel J.J., Lee W., Yuan J., Wong P., Ho T.S. (2015). Mutational Landscape Determines Sensitivity to PD-1 Blockade in Non–Small Cell Lung Cancer. Science.

[19] Hellmann M.D., Ciuleanu T.-E., Pluzanski A., Lee J.S., Otterson G.A., Audigier-Valette C., Minenza E., Linardou H., Burgers S., Salman P. (2018). Nivolumab plus Ipilimumab in Lung Cancer with a High Tumor Mutational Burden. N. Engl. J. Med..

[20] Miao D., Margolis C.A., Vokes N.I., Liu D., Taylor-Weiner A., Wankowicz S.M., Adeegbe D., Keliher D., Schilling B., Tracy A. (2018). Genomic Correlates of Response to Immune Checkpoint Blockade in Microsatellite-Stable Solid Tumors. Nat. Genet..

[21] Samstein R.M., Lee C.-H., Shoushtari A.N., Hellmann M.D., Shen R., Janjigian Y.Y., Barron D.A., Zehir A., Jordan E.J., Omuro A. (2019). Tumor Mutational Load Predicts Survival after Immunotherapy across Multiple Cancer Types. Nat. Genet..

[22] Gandara D.R., Paul S.M., Kowanetz M., Schleifman E., Zou W., Li Y., Rittmeyer A., Fehrenbacher L., Otto G., Malboeuf C. (2018). Blood-Based Tumor Mutational Burden as a Predictor of Clinical Benefit in Non-Small-Cell Lung Cancer Patients Treated with Atezolizumab. Nat. Med..

[23] Zehir A., Benayed R., Shah R.H., Syed A., Middha S., Kim H.R., Srinivasan P., Gao J., Chakravarty D., Devlin S.M. (2017). Mutational Landscape of Metastatic Cancer Revealed from Prospective Clinical Sequencing of 10,000 Patients. Nat. Med..

[24] Liu J., Lichtenberg T., Hoadley K.A., Poisson L.M., Lazar A.J., Cherniack A.D., Kovatich A.J., Benz C.C., Levine D.A., Lee A.V. (2018). An Integrated TCGA Pan-Cancer Clinical Data Resource to Drive High-Quality Survival Outcome Analytics. Cell.

[25] Mayakonda A., Lin D.-C., Assenov Y., Plass C., Koeffler H.P. (2018). Maftools: Efficient and Comprehensive Analysis of Somatic Variants in Cancer. Genome Res..

[26] Liu D., Liu X., Xing M. (2012). Epigenetic Genes Regulated by the BRAFV600E Signaling Are Associated with Alterations in the Methylation and Expression of Tumor Suppressor Genes and Patient Survival in Melanoma. Biochem. Biophys. Res. Commun..

[27] Gabriele M., Lopez Tobon A., D’Agostino G., Testa G. (2018). The Chromatin Basis of Neurodevelopmental Disorders: Rethinking Dysfunction along the Molecular and Temporal Axes. Prog. Neuro-Psychopharmacol. Biol. Psychiatry.

[28] Mossink B., Negwer M., Schubert D., Nadif Kasri N. (2021). The Emerging Role of Chromatin Remodelers in Neurodevelopmental Disorders: A Developmental Perspective. Cell Mol. Life Sci..

[29] Pitroda S.P., Pashtan I.M., Logan H.L., Budke B., Darga T.E., Weichselbaum R.R., Connell P.P. (2014). DNA Repair Pathway Gene Expression Score Correlates with Repair Proficiency and Tumor Sensitivity to Chemotherapy. Sci. Transl. Med..

[30] Newman A.M., Liu C.L., Green M.R., Gentles A.J., Feng W., Xu Y., Hoang C.D., Diehn M., Alizadeh A.A. (2015). Robust Enumeration of Cell Subsets from Tissue Expression Profiles. Nat. Methods.

[31] Liu D., Vadgama J., Wu Y. (2021). Basal-like Breast Cancer with Low TGFβ and High TNFα Pathway Activity Is Rich in Activated Memory CD4 T Cells and Has a Good Prognosis. Int. J. Biol. Sci..

[32] Liu D. (2020). AR Pathway Activity Correlates with AR Expression in a HER2-Dependent Manner and Serves as a Better Prognostic Factor in Breast Cancer. Cell Oncol..

[33] Liberzon A., Birger C., Thorvaldsdóttir H., Ghandi M., Mesirov J.P., Tamayo P. (2015). The Molecular Signatures Database (MSigDB) Hallmark Gene Set Collection. Cell Syst..

[34] Heeke S., Benzaquen J., Hofman V., Long-Mira E., Lespinet V., Bordone O., Marquette C.-H., Delingette H., Ilié M., Hofman P. (2020). Comparison of Three Sequencing Panels Used for the Assessment of Tumor Mutational Burden in NSCLC Reveals Low Comparability. J. Thorac. Oncol..

[35] Alborelli I., Leonards K., Rothschild S.I., Leuenberger L.P., Savic Prince S., Mertz K.D., Poechtrager S., Buess M., Zippelius A., Läubli H. (2020). Tumor Mutational Burden Assessed by Targeted NGS Predicts Clinical Benefit from Immune Checkpoint Inhibitors in Non-Small Cell Lung Cancer. J. Pathol..

[36] Niu X., Zhang T., Liao L., Zhou L., Lindner D.J., Zhou M., Rini B., Yan Q., Yang H. (2012). The von Hippel-Lindau Tumor Suppressor Protein Regulates Gene Expression and Tumor Growth through Histone Demethylase JARID1C. Oncogene.

[37] Astolfi A., Fiore M., Melchionda F., Indio V., Bertuccio S.N., Pession A. (2019). BCOR Involvement in Cancer. Epigenomics.

[38] Gut P., Verdin E. (2013). The Nexus of Chromatin Regulation and Intermediary Metabolism. Nature.

[39] Li X., Wenes M., Romero P., Huang S.C.-C., Fendt S.-M., Ho P.-C. (2019). Navigating Metabolic Pathways to Enhance Antitumour Immunity and Immunotherapy. Nat. Rev. Clin. Oncol..

[40] Zelenay S., van der Veen A.G., Böttcher J.P., Snelgrove K.J., Rogers N., Acton S.E., Chakravarty P., Girotti M.R., Marais R., Quezada S.A. (2015). Cyclooxygenase-Dependent Tumor Growth through Evasion of Immunity. Cell.

[41] Wang G.G., Allis C.D., Chi P. (2007). Chromatin Remodeling and Cancer, Part II: ATP-Dependent Chromatin Remodeling. Trends Mol. Med..

[42] Centore R.C., Sandoval G.J., Soares L.M.M., Kadoch C., Chan H.M. (2020). Mammalian SWI/SNF Chromatin Remodeling Complexes: Emerging Mechanisms and Therapeutic Strategies. Trends Genet..

[43] Braun D.A., Ishii Y., Walsh A.M., Van Allen E.M., Wu C.J., Shukla S.A., Choueiri T.K. (2019). Clinical Validation of PBRM1 Alterations as a Marker of Immune Checkpoint Inhibitor Response in Renal Cell Carcinoma. JAMA Oncol..

