# Supplementary material for: Mutations in KMT2C, BCOR and KDM5C Predict Response to Immune Checkpoint Blockade Therapy in Non-Small Cell Lung Cancer
Source: Cancers (Basel). 2022 Jun 6;14(11):2816. doi: 10.3390/cancers14112816 (PMC9179442; doi:10.3390/cancers14112816)
Supplement: Supplementary file 1 [file cancers-14-02816-s001.zip › cancers-1706114-supplementary.pdf]

cancers

MDPI

Supplementary Material

Mutations in *KMT2C*, *BCOR* and *KDM5C* Predict Response to Immune Checkpoint Blockade Therapy in Non-Small Cell Lung Cancer.

Dingxie Liu, Jonathan Benzaquen, Luc G. T. Morris, Marius Ilié, and Paul Hofman

Table S1. Demographic features of patients in seven NSCLC cohorts.

Characteristic	Hellmann2018 Case No. (%)	Miao2018 Case No. (%)	Rizvi2018 Case No. (%)	Samstein2019 Case No. (%)	In-house cohort Case No. (%)	POPLAR/OAK Case No. (%)	Zehir 2017 Case No. (%)	TCGA LUAD Case No. (%)	TCGA LUSC Case No. (%)
Median Age (range)	66 (42–87)	62.5 (41–80)	66 (22–92)	67 (31–90)	61 (38–83)	64 (42–82)	NA	66 (33–88)	68 (39–90)
Sex									
Female	38 (50.67%)	36 (57.14%)	122 (50.83%)	178 (51.74%)	5 (13.00%)	53 (32.00%)	253 (57.50%)	276 (53.59%)	128 (26.02%)
Male	37 (49.33%)	27 (42.86%)	118 (49.17%)	166 (48.26%)	33 (87.00%)	113 (68.00%)	187 (42.50%)	239 (46.41%)	364 (73.98%)
NSCLC Histology									
Adenocarcinoma	75 (100.00%)	54 (88.52%)	186 (84.55%)	266 (85.81%)	23 (61.00%)	NA	379 (93.12%)	515 (100.00%)	0
Squamous	0	7 (11.48%)	34 (15.45%)	44 (14.19%)	11 (29.00%)	47 (28.00%)	28 (6.88%)	0	492 (100.00%)
Other	0	2 (3.28%)	20 (9.09%)	34 (10.97%)	4 (11.00%)	NA	33 (8.11%)	0	0
ICB targeting									
PD-1/PD-L1	0	63 (100.00%)	206 (85.83%)	323 (93.90%)	38 (100.00%)	166 (100.00%)	NE	NE	NE
PD-1/PD-L1 & CTLA-4	75 (100.00%)	0 (0.00%)	34 (14.17%)	21 (6.10%)	0 (0.00%)	0 (0.00%)	NE	NE	NE
Response to ICB									
CR/PR	24 (58.54%)	19 (47.50%)	49 (31.21%)	NA		23 (16.00%)	NE	NE	NE
SD	17 (41.46%)	21 (52.50%)	108 (68.79%)	NA		83 (58.00%)	NE	NE	NE
PD	27 (65.85%)	23 (57.50%)	83 (52.87%)	NA		38 (26.00%)	NE	NE	NE
Clinical benefit for ICB									

NCB	38 (50.67%)	33 (52.38%)	158 (69.60%)	NA	26 (68.00%)	125 (75.00%)	NE	NE	NE
DCB	37 (49.33%)	30 (47.62%)	69 (30.40%)	NA	12 (32.00%)	41 (25.00%)	NE	NE	NE

* Only samples with sequencing data were counted in. ** Besides the initial 56 NSCLC cases, Miao 2018 cohort also includes the 7 non-overlapped cases from Rizvi 2015 cohort. *** Only immunotherapy-treated NSCLC cases with Median_exon_coverage >2000 in POPLAR/OAK cohort were counted in. **** Only Non-immunotherapy-treated NSCLC cases in Zehir 2017 cohort were counted in.

Table S2. Mutation rates of *KMT2C*, *BCOR* and *KDM5C* in NSCLC subgroups.

**Subgroup**	**Subgroup**	**Case number**	**Mutation Number (Percentage)**			
			**KMT2C**	**BCOR**	**KDM5C**	***KMT2C/BCOR/KDM5C***
Hellmann 2018	Male	37	3 (8.11%)	0 (0.00%)	1 (2.70%)	4 (10.81%)
	Female	38	5 (13.16%)	3 (7.89%)	1 (2.63%)	7 (18.42%)
	Age ≤55	14	2 (14.29%)	0 (0.00%)	0 (0.00%)	2 (14.29%)
	Age 55–65	23	2 (8.70%)	1 (4.35%)	0 (0.00%)	2 (8.70%)
	Age >65	38	4 (10.53%)	2 (5.26%)	2 (5.26%)	7 (18.42%)
Miao 2018	Male	27	1 (3.70%)	0 (0.00%)	0 (0.00%)	1 (3.70%)
	Female	36	5 (13.89%)	3 (8.33%)	1 (2.78%)	8 (22.22%)
	Age ≤55	8	1 (12.50%)	0 (0.00%)	0 (0.00%)	1 (12.50%)
	Age 55–65	25	4 (16.00%)	1 (4.00%)	1 (4.00%)	5 (20.00%)
	Age >65	15	0 (0.00%)	0 (0.00%)	0 (0.00%)	0 (0.00%)
Rizvi 2018	Male	118	11 (9.32%)	5 (4.24%)	7 (5.93%)	21 (17.80%)
	Female	122	14 (11.48%)	6 (4.92%)	3 (2.46%)	21 (17.21%)
	Age ≤ 55	49	7 (14.29%)	2 (4.08%)	2 (4.08%)	9 (18.37%)
	Age 55–65	67	9 (13.43%)	4 (5.97%)	3 (4.48%)	15 (22.39%)
	Age > 65	124	9 (7.26%)	5 (4.03%)	5 (4.03%)	18 (14.52%)
Samstein 2019	Male	166	15 (9.04%)	3 (1.81%)	7 (4.22%)	21 (12.65%)
	Female	178	20 (11.24%)	13 (7.30%)	2 (1.12%)	30 (16.85%)
	Age ≤55	56	10 (17.86%)	5 (8.93%)	1 (1.79%)	10 (17.86%)
	Age 55–65	100	8 (8.00%)	5 (5.00%)	3 (3.00%)	15 (15.00%)
	Age >65	188	17 (9.04%)	6 (3.19%)	5 (2.66%)	26 (13.83%)
Zehir 2017	Male	187	8 (4.28%)	3 (1.60%)	6 (3.21%)	13 (6.95%)
	Female	253	18 (7.11%)	7 (2.77%)	6 (2.37%)	26 (10.28%)
TCGA LUAD	MALE	239	29 (12.13%)	5 (2.09%)	11 (4.60%)	38 (15.90%)
	FEMALE	276	23 (8.33%)	17 (6.16%)	5 (1.81%)	35 (12.68%)
	Age ≤55	79	10 (12.66%)	6 (7.59%)	2 (2.53%)	15 (18.99%)
	Age 55–65	159	18 (11.32%)	11 (6.92%)	4 (2.52%)	26 (16.35%)
	Age >65	258	23 (8.91%)	5 (1.94%)	10 (3.88%)	31 (12.02%)
	Stage I	277	29 (10.47%)	15 (5.42%)	6 (2.17%)	42 (15.16%)
	Stage II	121	15 (12.40%)	4 (3.31%)	4 (3.31%)	18 (14.88%)
	Stage III	84	6 (7.14%)	2 (2.38%)	3 (3.57%)	8 (9.52%)
	Stage IV	26	2 (7.69%)	0 (0.00%)	3 (11.54%)	4 (15.38%)
TCGA LUSC	MALE	364	46 (12.64%)	7 (1.92%)	9 (2.47%)	55 (15.11%)
	FEMALE	128	13 (10.16%)	7 (5.47%)	0 (0.00%)	18 (14.06%)
	Age ≤55	45	2 (4.44%)	1 (2.22%)	1 (2.22%)	4 (8.89%)
	Age 55–65	141	21 (14.89%)	5 (3.55%)	4 (2.84%)	28 (19.86%)
	Age >65	297	36 (12.12%)	8 (2.69%)	4 (1.35%)	41 (13.80%)
	Stage I	240	26 (10.83%)	4 (1.67%)	5 (2.08%)	31 (12.92%)
	Stage II	158	21 (13.29%)	6 (3.80%)	2 (1.27%)	25 (15.82%)
	Stage III	83	9 (10.84%)	3 (3.61%)	2 (2.41%)	14 (16.87%)
	Stage IV	7	2 (28.57%)	1 (14.29%)	0 (0.00%)	2 (28.57%)

*Tumor stage information is only available for TCGA cohorts.

Table S3. Hallmark and KEGG pathway gene sets that are suppressed or activated by BCOR/KMT2C/KDM5C mutations.

TCGA Cohort	Hallmark/KEGG Gene Sets	Size	NES	Adj. p-val	FDR q-val
LUAD	KEGG_ASCORBATE_AND_ALDARATE_METABOLISM	68	-2.24	4.47E-05	3.71E-05
LUAD	KEGG_STARCH_AND_SUCROSE_METABOLISM	59	-2.26	8.52E-05	7.06E-05
LUAD	KEGG_PENTOSE_AND_GLUCURONATE_INTERCONVERSIONS	66	-2.20	8.52E-05	7.06E-05
LUAD	KEGG_COMPLEMENT_AND_COAGULATION_CASCADES	134	-1.93	8.52E-05	7.06E-05
LUAD	KEGG_DRUG_METABOLISM_OTHER_ENZYMES	199	-1.77	1.94E-04	1.61E-04
LUAD	KEGG_LINOLEIC_ACID_METABOLISM	199	-1.76	2.13E-04	1.76E-04
LUAD	KEGG_ARACHIDONIC_ACID_METABOLISM	36	-2.27	2.14E-04	1.77E-04
LUAD	HALLMARK_XENOBIOTIC_METABOLISM	37	-2.23	4.88E-04	4.04E-04
LUAD	HALLMARK_XENOBIOTIC_METABOLISM	48	-2.10	4.93E-04	4.08E-04
LUAD	KEGG_DRUG_METABOLISM_CYTOCHROME_P450	21	-2.18	1.22E-03	1.01E-03
LUAD	KEGG_METABOLISM_OF_XENOBIOTICS_BY_CYTOCHROME_P450	44	-1.98	1.81E-03	1.50E-03
LUAD	KEGG_RETINOL_METABOLISM	24	-2.18	2.05E-03	1.70E-03
LUAD	HALLMARK_INFLAMMATORY_RESPONSE	66	-1.80	8.86E-03	7.34E-03
LUAD	KEGG_ARACHIDONIC_ACID_METABOLISM	46	-1.88	0.01	0.01
LUAD	KEGG_ABC_TRANSPORTERS	27	-1.99	0.01	0.01
LUAD	KEGG_CYTOKINE_CYTOKINE_RECEPTOR_INTERACTION	54	-1.80	0.01	0.01
LUAD	KEGG_COMPLEMENT_AND_COAGULATION_CASCADES	198	-1.47	0.01	0.01
LUSC	KEGG_LINOLEIC_ACID_METABOLISM	198	-2.03	1.18E-08	9.53E-09
LUSC	KEGG_DRUG_METABOLISM_OTHER_ENZYMES	70	-2.17	2.30E-07	1.86E-07
LUSC	KEGG_HEMATOPOIETIC_CELL_LINEAGE	68	-2.15	3.54E-07	2.87E-07
LUSC	HALLMARK_BILE_ACID_METABOLISM	63	-2.07	2.42E-06	1.96E-06
LUSC	HALLMARK_FATTY_ACID_METABOLISM	200	-1.85	2.85E-06	2.31E-06
LUSC	HALL-MARK_TNFA_SIGNALING_VIA_NFKB	53	-2.05	1.88E-05	1.52E-05
LUSC	KEGG_CHEMOKINE_SIGNALING_PATHWAY	44	-2.06	6.60E-05	5.35E-05
LUSC	HALLMARK_ANDROGEN_RESPONSE	245	-1.71	9.56E-05	7.75E-05
LUSC	KEGG_PORPHYRIN_AND_CHLOROPHYLL_METABOLISM	66	-1.94	1.55E-04	1.26E-04
LUSC	HALLMARK_HYPOXIA	26	-2.04	2.10E-04	1.71E-04
LUSC	KEGG_PENTOSE_AND_GLUCURONATE_INTERCONVERSIONS	50	-1.92	2.05E-03	1.66E-03
LUAD	HALLMARK_G2M_CHECKPOINT	200	2.20	1.18E-08	9.74E-09
LUAD	HALLMARK_E2F_TARGETS	200	2.28	1.18E-08	9.74E-09
LUAD	KEGG_CELL_CYCLE	124	1.80	3.62E-04	3.00E-04
LUAD	HALLMARK_SPERMATOGENESIS	120	1.81	4.95E-04	4.10E-04
LUAD	HALLMARK_MITOTIC_SPINDLE	199	1.70	5.37E-04	4.45E-04
LUSC	HALL-	97	2.54	1.18E-08	9.53E-09

	MARK_INTERFERON_ALPHA_RESPONSE				
LUSC	HALL-MARK_INTERFERON_GAMMA_RESPONSE	200	1.59	1.08E-03	8.79E-04
LUSC	KEGG_RIBOSOME	87	1.63	0.01	0.01

*NES: Normalized enrichment score. ** Adj.p-val: BH-adjusted p-value.

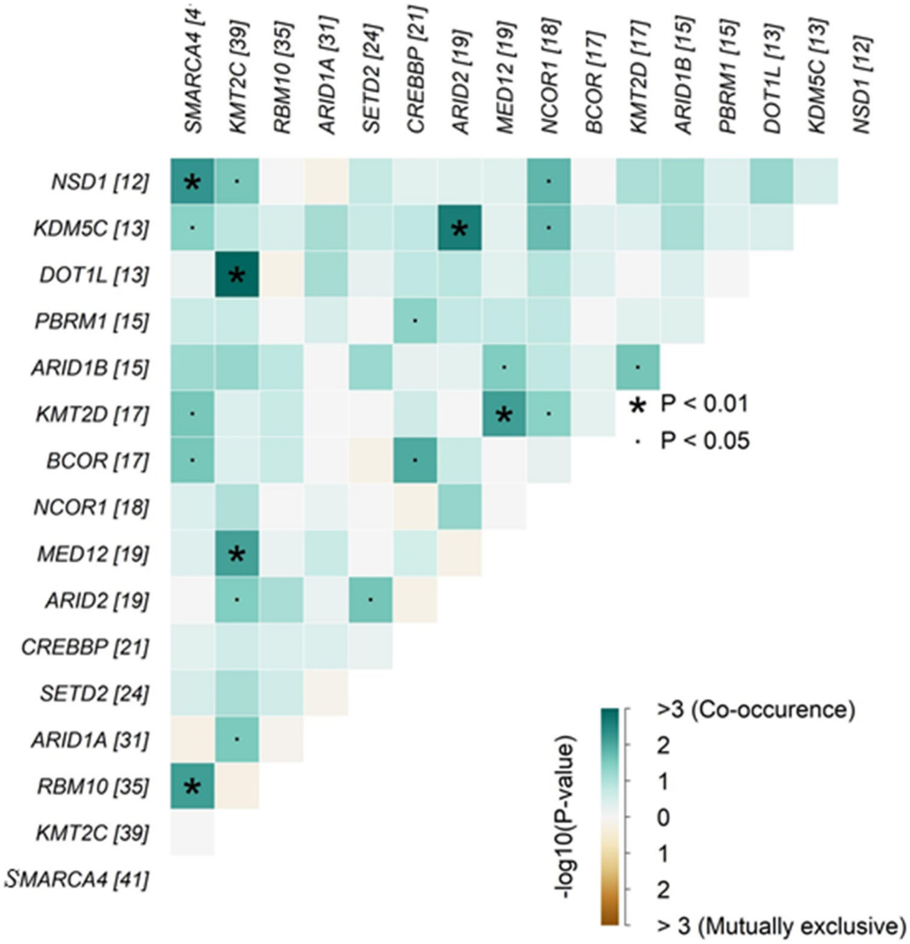

Figure S1. Heatmap displaying consistency and exclusivity correlations among 15 chromatin remodeling-related genes in NSCLC. The color scale represents -log10 p-value. The cadet blue indicates co-occurrence and the copper indicates mutual exclusion.

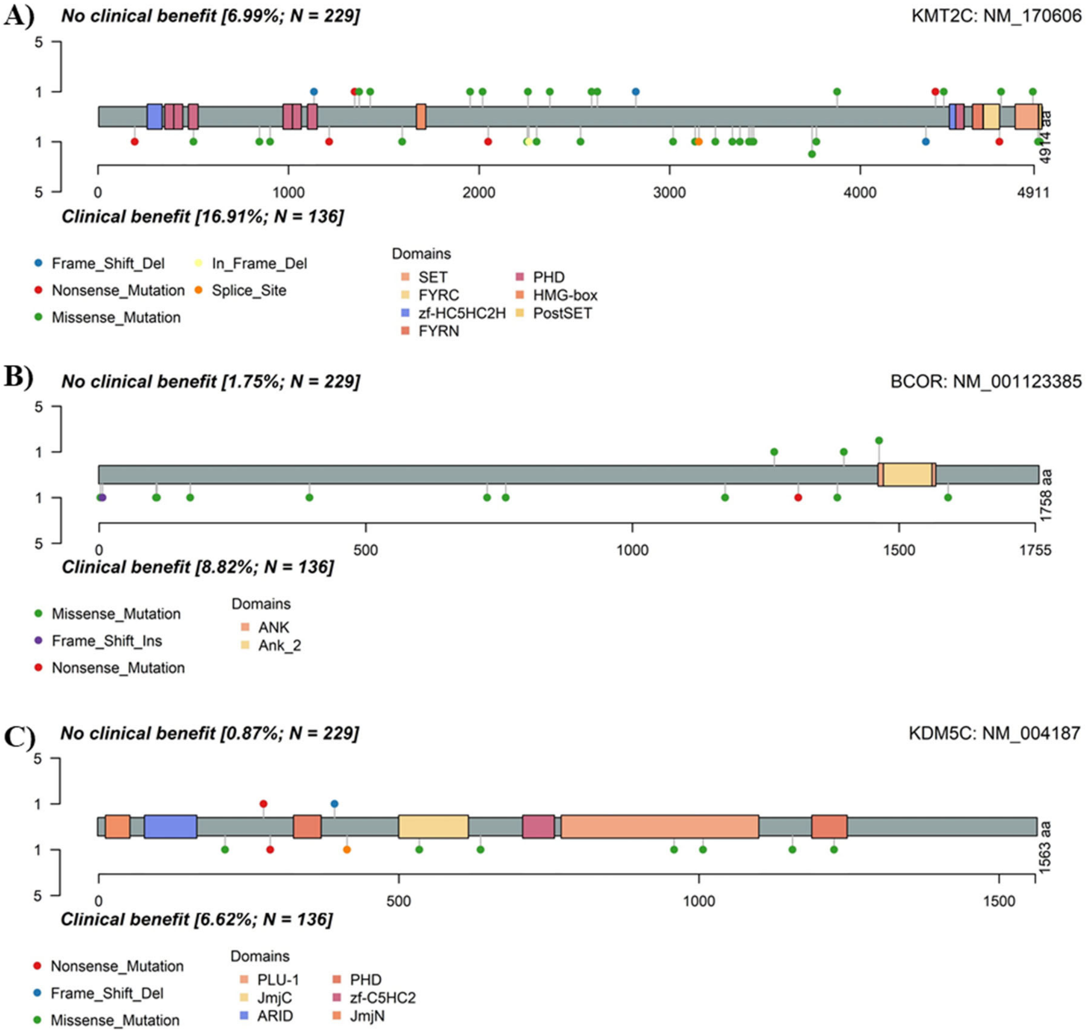

Figure S2. Lollipop plot displaying mutation distribution and protein domains for three chromatin remodeling-related genes in NSCLC. **A**) *KMT2C*. **B**) *BCOR*. **C**) *KDM5C*. Somatic mutation rate and transcript names are indicated by plot title and subtitle, respectively. Mutations in patients with no durable benefit (NDB) and Durable clinical benefit (DCB) were shown in the upper and bottom sides, respectively.

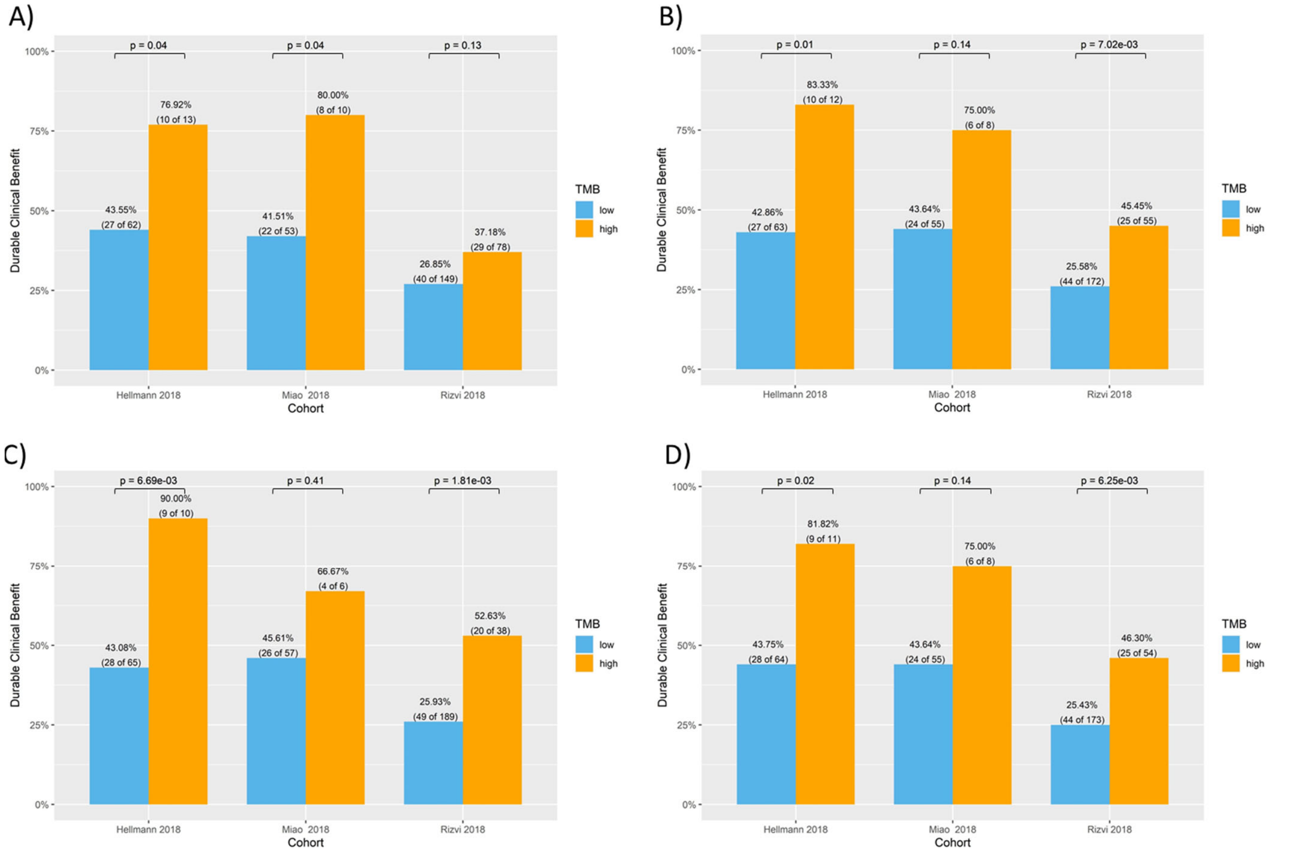

Figure S3. Comparison of DCB rates between NSCLC patients with low and high tumor mutation burden (TMB) levels. Three Immune checkpoint blockade (ICB) therapy cohorts were tested as indicated. TMB-high and TMB-low subgroups were stratified based on different cutoff values. **A)** Cutoff at 10 mutations/Mb. **B)** Cutoff at 12 mutations/Mb. **C)** Cutoff at 15 mutations/Mb. **D)** Cutoff at top 20% TMB value within each cohort as cutoff.

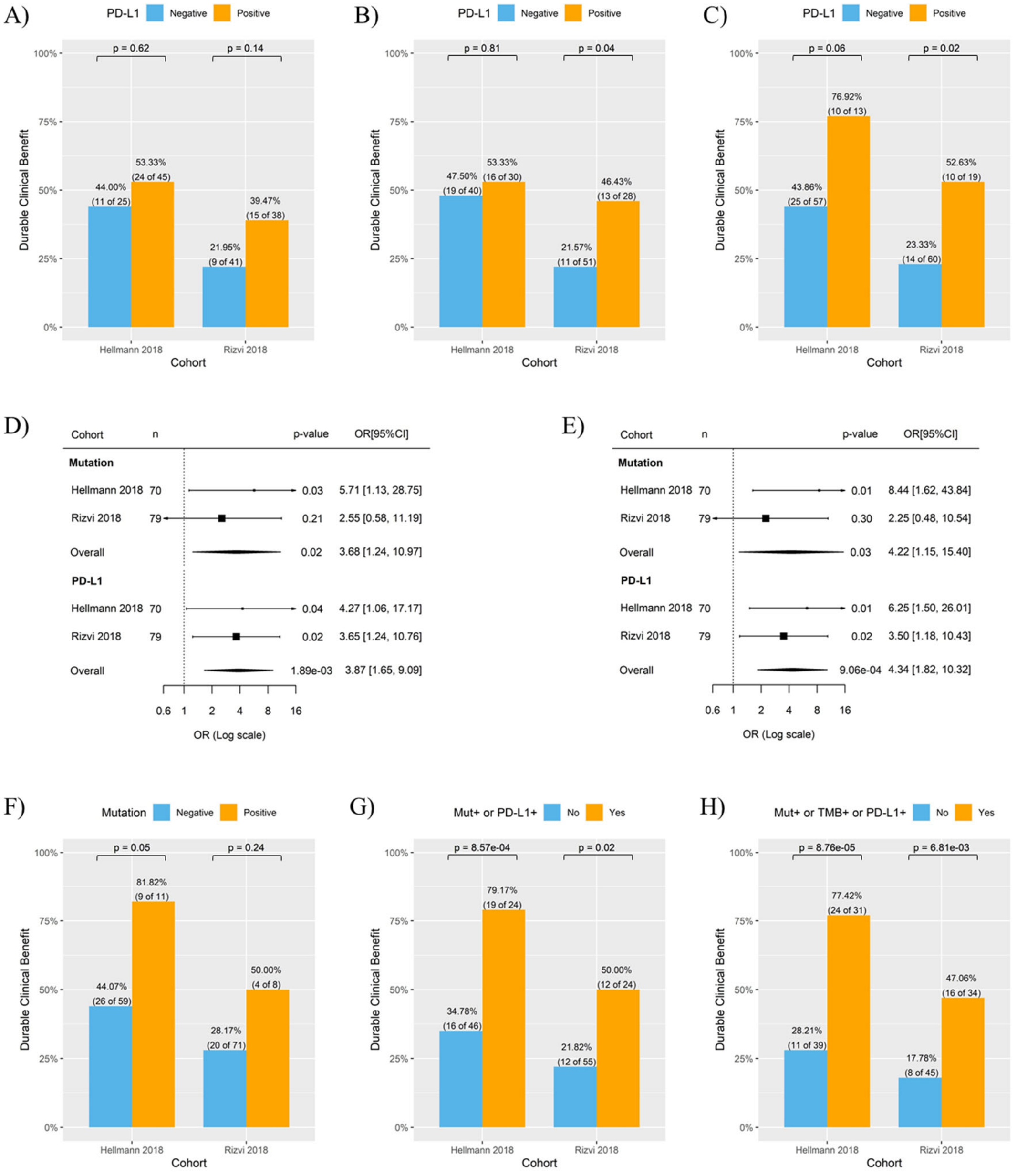

Figure S4. Association of PD-L1 expression, alone or in combination, with NSCLC response to ICB therapy. Two ICB therapy cohorts that were annotated with PD-L1 expression data were tested as indicated. **A–C)** Histogram showing the association of PD-L1 expression with NSCLC reScheme 1. staining were set as 1% **(A)**, 10% **(B)** or 30% **(C)**. **D–E)** Univariate **(D)** and multivariate **(E)** logistic regression analysis of the correlations of ICB response with PD-L1 expression and *KMT2C*/*BCOR*/*KDM5C* mutations. Both *KMT2C*/*BCOR*/*KDM5C* mutations (indicated as combination in the plot) and PD-L1 were used as dichotomous variables in regression analysis. The cut-off value used for the minimum percentage of cells positive for PD-L1 staining was set as 30%. F) Histogram showing the association of *KMT2C*/*BCOR*/*KDM5C* mutations with ICB response in NSCLC annotated with PD-L1 expression data. **G)** Histogram showing the synergistic interactions of PD-L1 expression with *KMT2C*/*BCOR*/*KDM5C* mutations on NSCLC response to ICB therapy. **G)** Histogram showing the synergistic interactions among *KMT2C*/*BCOR*/*KDM5C* mutations, PD-L1 expression and TMB on NSCLC response to ICB therapy. No: sample with wild-type *KMT2C*/*BCOR*/*KDM5C*, negative PD-L1 expression, and low TMB; Yes: sample with mutant *KMT2C*/*BCOR*/*KDM5C*, positive PD-L1 expression or low TMB.

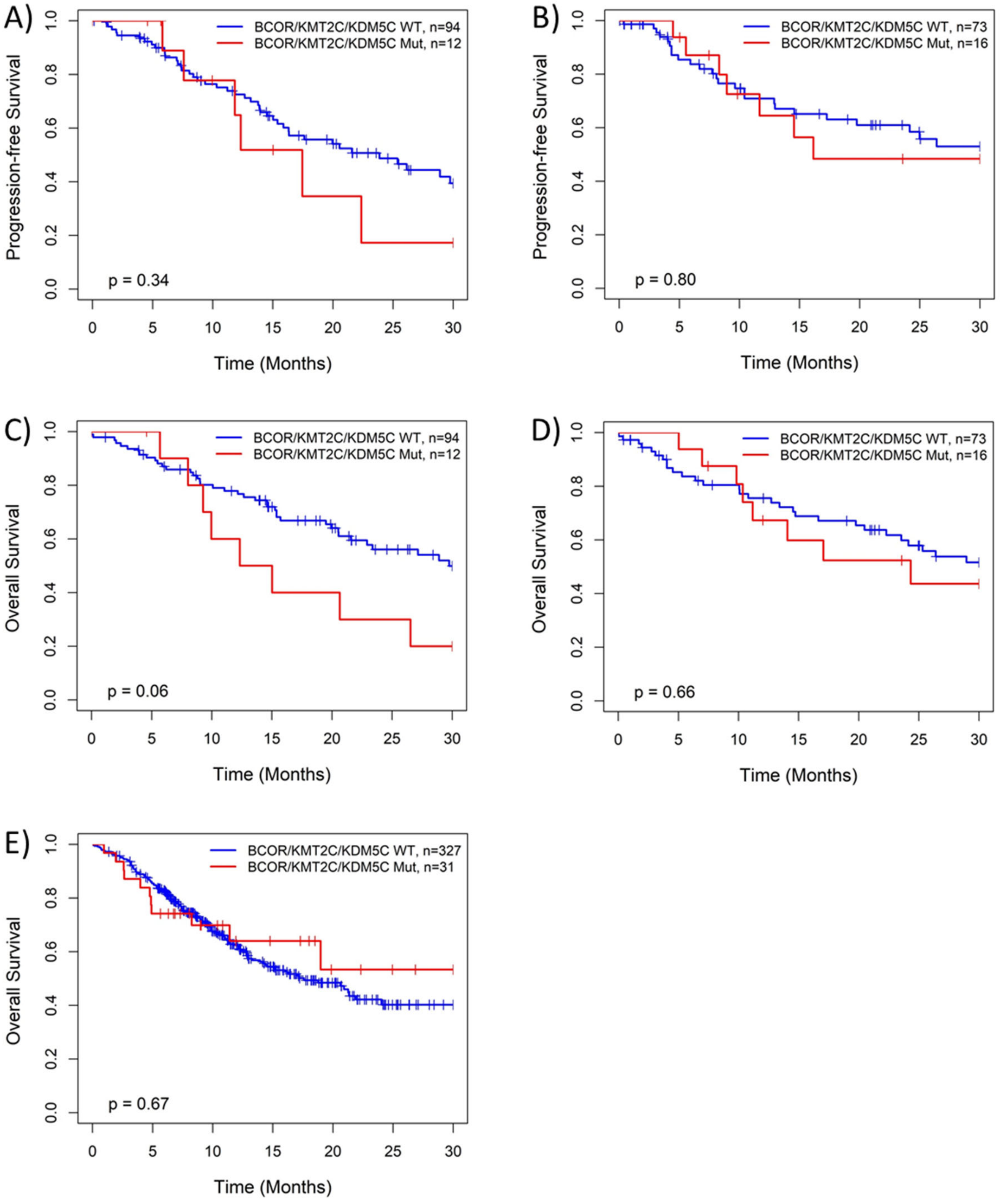

Figure S5. Kaplan–Meier analysis of the associations of *KMT2C*/*BCOR*/*KDM5C* mutations with prognosis of NSCLC without ICB therapy record. The cases with immunotherapy records in TCGA cohorts were excluded here in survival analysis. Since NSCLCs in the ICB cohorts tested in this study are all advanced tumors, here we only analyzed stage III and IV tumors in TCGA cohorts. **A**) Association with PFS in TCGA LUAD cohort. **B**) Association with PFS in TCGA LUSC cohort. **C**) Association with OS in TCGA LUAD cohort. **D**) Association with OS in TCGA LUSC cohort. **E**) Association with OS in patients without ICB therapy record in Zehir 2017 cohort. In Zehir 2017 cohort, there are only 28 LUSC samples and none of these samples carry mutations in BCOR, KMT2C or KDM5C. Therefore, only LUAD samples were analyzed here.

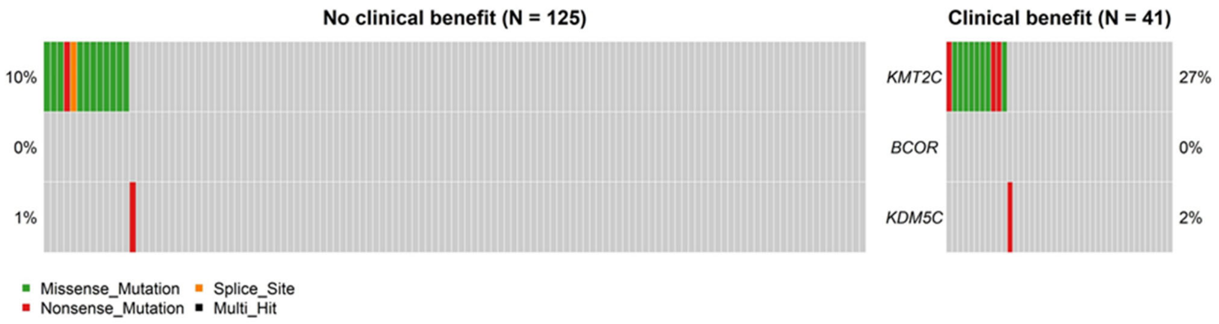

Figure S6. Oncoplot displaying the somatic mutations of *KMT2C*, *BCOR* and *KDM5C* in NSCLC of OAK/POPLAR cohort. Each column represents a sample. The numbers shown at left and right sides represent the gene mutation rates.

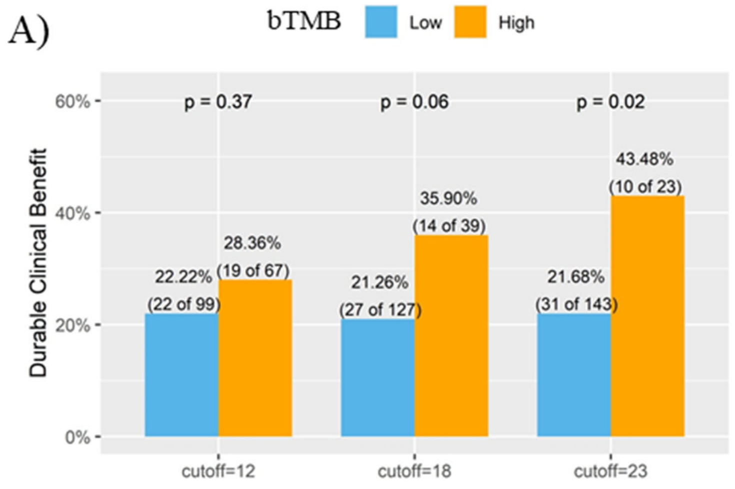

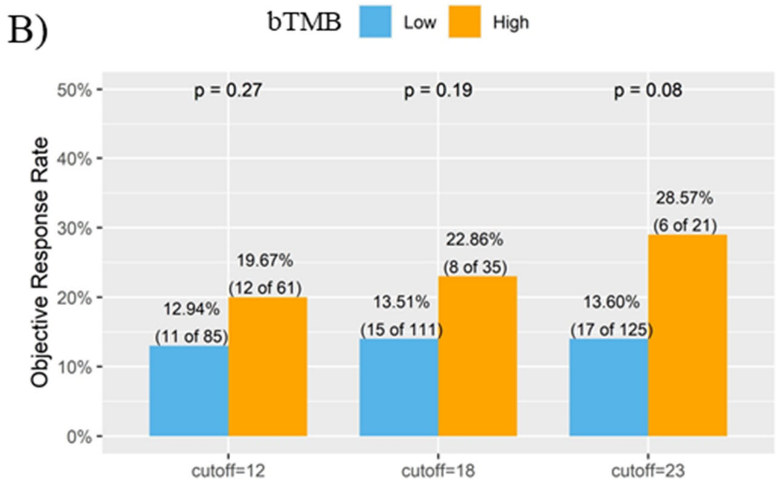

Figure S7. Comparison of ICB response between NSCLC patients with low and high blood TMB (bTMB) levels**.** DCB **(A)** and ORR **(B)** rates were compared between NSCLC subgroups in OAK/POPLAR cohort. Subgroups of bTMB-high and bTMB-low were stratified based on different cutoff values, including 12, 18 and 23 mutations/Mb as indicated.

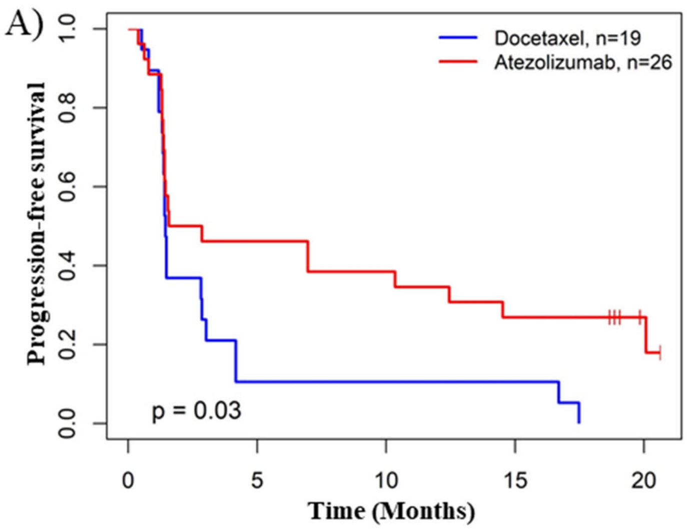

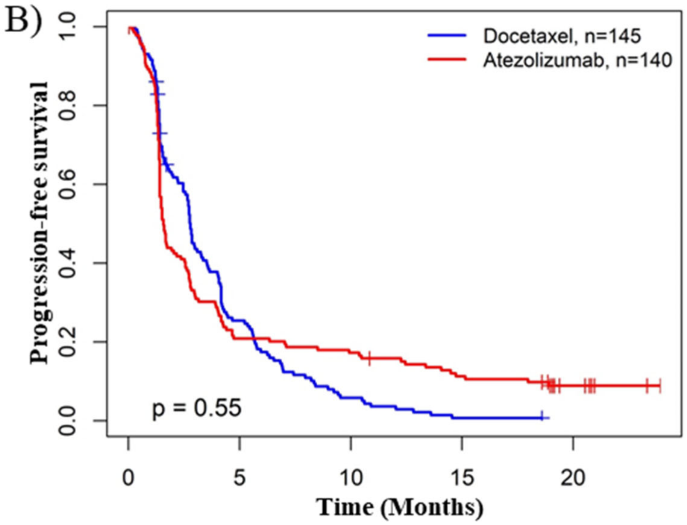

Figure S8. Comparison of the therapeutic effects of atezolizumab and docetaxel in different NSCLC subgroups of OAK/POPLAR cohort. **A**) NSCLC patients carrying KMT2C/BCOR/KDM5C mutations. **B**) NSCLC patients without KMT2C/BCOR/KDM5C mutations. Survival benefits from atezolizumab and docetaxel treatment were compared using Kaplan-Meier analysis a anxel treatment were compared using Kaplan-Meier analysis.

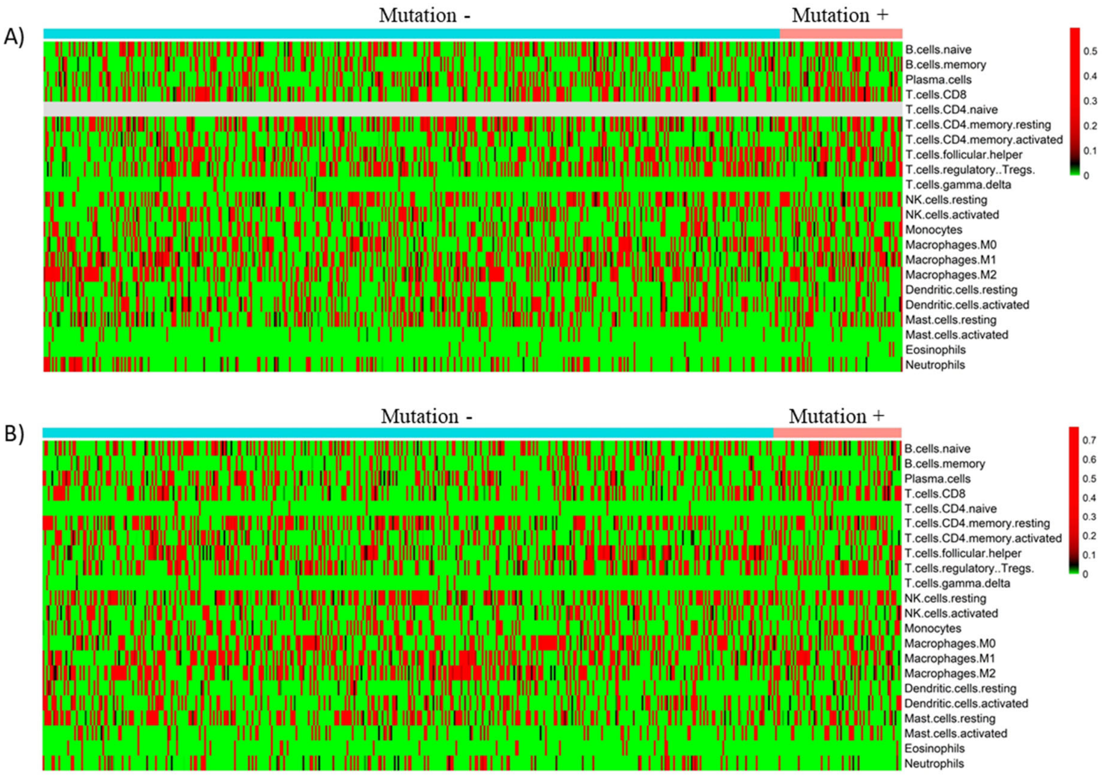

Figure S9. Associations of *KMT2C*/*BCOR*/*KDM5C* mutations with immune cell subsets infiltration in NSCLC**. A)** TCGA LUAD cohort. **B)** TCGA LUSC cohort. The fractions of 22 immune cell subsets were computed using CIBERSORT algorithm. The heatmap was used to depict relative immune cell subset levels across two groups of samples with or without *KMT2C*/*BCOR*/*KDM5C* mutations. Each row represents one immune cell subset. Each column represents one NSCLC sample. Mutation -: *KMT2C*/*BCOR*/*KDM5C* wild-type; Mutation +: Mutation in *KMT2C*/*BCOR*/*KDM5C*.

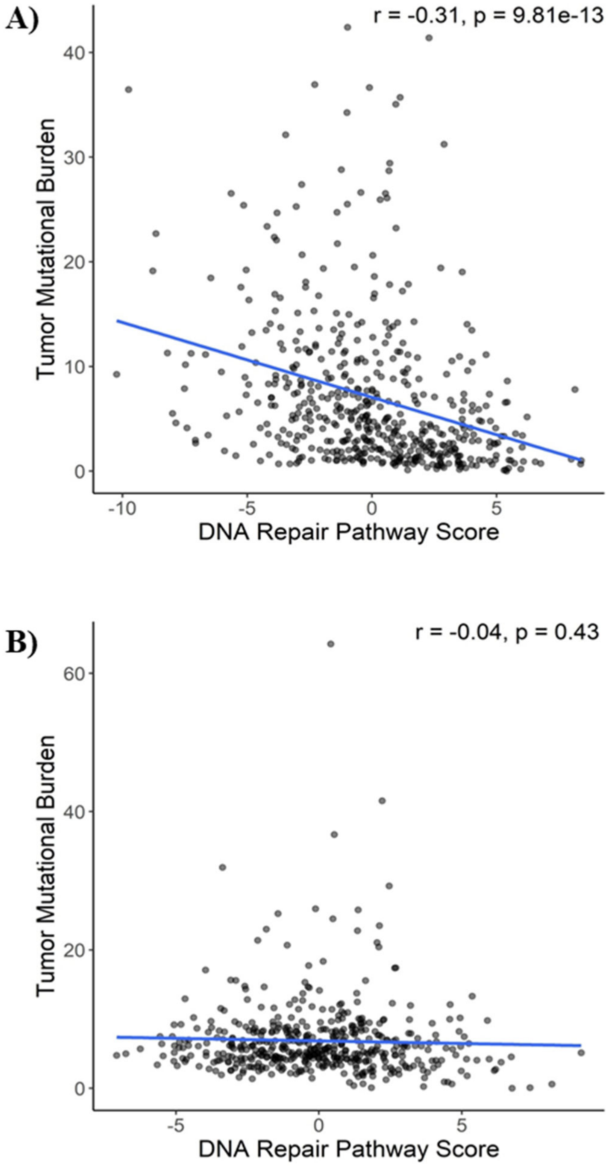

Figure S10. Correlation between DNA repair pathway score and TMB level in TCGA NSCLC cohorts**. A)** TCGA LUAD cohort. **B**) LUSC cohort. Each dot represents one sample. The trendline was generated by linear regression.

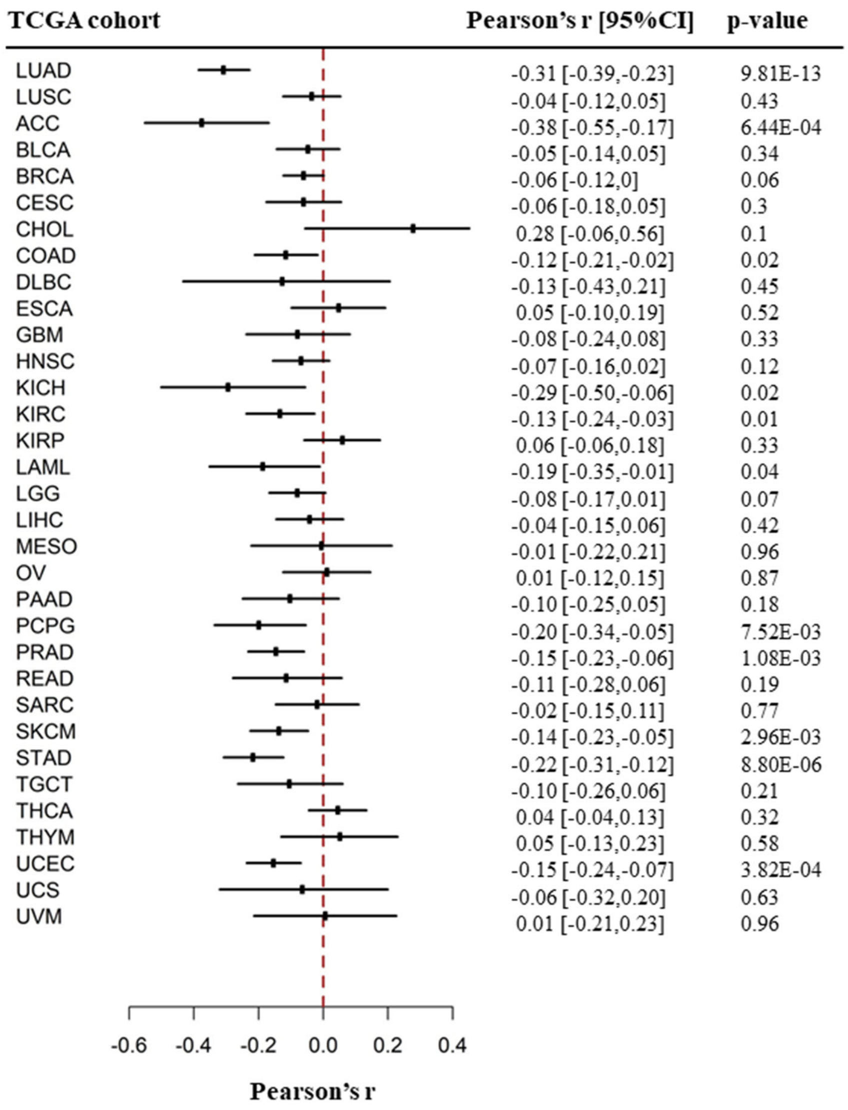

Figure S11. Correlation between DNA repair pathway score and TMB level in TCGA pan-cancer cohorts. The Pearson's r values are shown in forest plots, in which the squares and horizontal lines represent the Pearson's r and 95% CI for the individual cohorts.

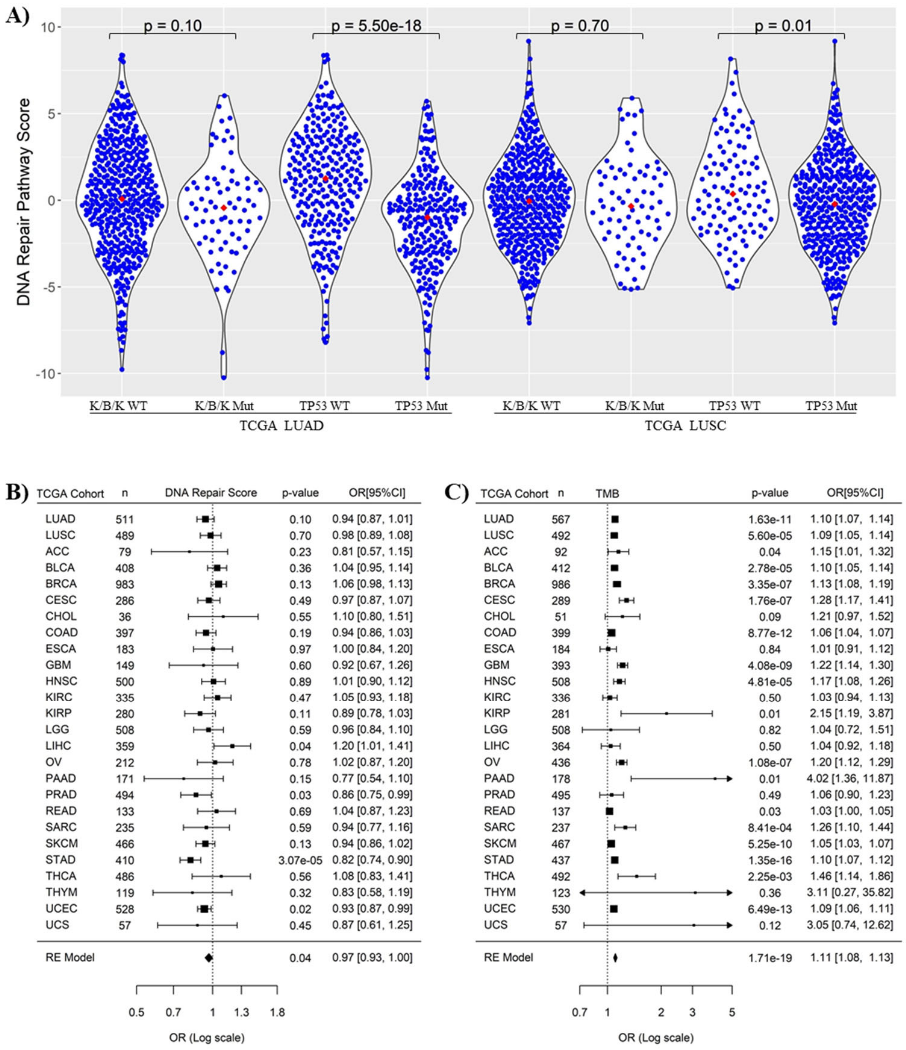

Figure S12. Association of *KMT2C*/*BCOR*/*KDM5C* mutations with DNA repair pathway score in NSCLC. **A)** Correlation between DNA repair pathway score and *KMT2C*/*BCOR*/*KDM5C* mutations in TCGA NSCLC cohorts. *TP53* mutation was used as positive control here. Each dot represents one sample and red dots represent median TMB values. K/B/K: *KMT2C*/*BCOR*/*KDM5C*. **B–C)** Logistic regression analysis of the correlation of *KMT2C*/*BCOR*/*KDM5C* mutations with DNA repair pathway scores **(B)** or TMB levels **(C)** in TCGA pan-cancer cohorts (mutant as event and wild-type as non-event). Only cohorts with event case ≥4 were included for regression analysis. DNA repair pathway scores and TMB levels were used as a continuous variable, and the random-effects model was used to calculate the overall effect.

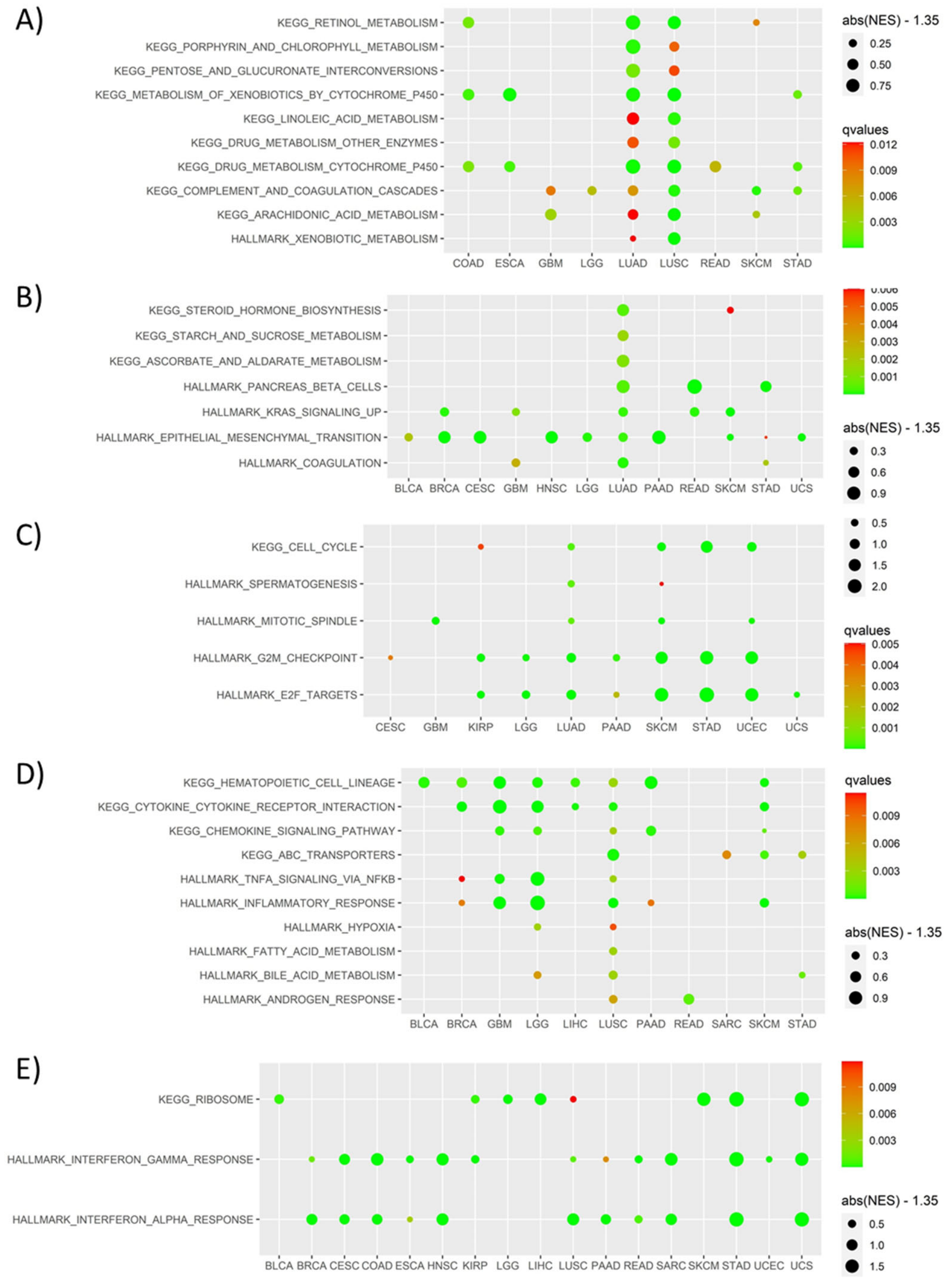

Figure S13. *KMT2C*/*BCOR*/*KDM5C* mutations and enrichment of Hallmark and KEGG pathway gene sets**.** Dot plot showing mutations of *KMT2C*/*BCOR*/*KDM5C*-related enrichment of Hallmark and KEGG gene sets in GSEA analysis. **A)** Gene sets suppressed by *KMT2C*/*BCOR*/*KDM5C* mutations in both LUAD and LUSC cohorts. **B–C)** Gene sets suppressed (**B**) or activated (**C**) by *KMT2C*/*BCOR*/*KDM5C* mutations in LUAD but not LUSC cohorts. **D**–**E**) Gene sets suppressed (**D**) or activated (**E**) by *KMT2C*/*BCOR*/*KDM5C* mutations in LUSC but not LUAD cohorts. Each node dot represents a gene set. The dot size represents the enrichment degree (shown as the absolute value of NES minus 1.35) of gene sets. The color intensity of dotes represents the FDR values of the gene set in GSEA analysis. Totally 21 TCGA pan-cancer, which are indicated in **Figure S12B,C**, were tested here. Several of the 21 TCGA cohorts were not shown in this figure because significant enrichment of the indicated gene sets was not observed in the TCGA cohort. NES: Normalized enrichment score.
